# Status, Trend, and Prospect of Global Farmland Abandonment Research: A Bibliometric Analysis

**DOI:** 10.3390/ijerph192316007

**Published:** 2022-11-30

**Authors:** Bo Liu, Wei Song, Qian Sun

**Affiliations:** 1 Key Laboratory of Land Surface Pattern and Simulation, Institute of Geographic Sciences and Natural Resources Research, Chinese Academy of Sciences, Beijing 100101, China; 2 School of Geomatics, Liaoning Technical University, Fuxin 123000, China; 3 Hebei Collaborative Innovation Center for Urban-Rural Integration Development, Shijiazhuang 050061, China; 4 National Research Center for Geoanalysis, China Geological Survey, Beijing 100037, China

**Keywords:** farmland abandonment, research progress, theme evolution, biodiversity, reclamation, bibliometric analysis

## Abstract

Farmland abandonment is one of the most important land use changes in the world today and crucial to the sustainable development of the global environment. The authors carried out extensive research on farmland abandonment from many perspectives, but, due to the variety of the research contents, rich research perspectives, and complex research objects, the current research in this field lacks comprehensiveness, objectivity, and systematization. In this study, the bibliometric R software packages bibliometrix and biblioshiny (K-Synth Srl, Naples, Italy) were used to analyze the development history and current situation of 896 articles on farmland abandonment in the Web of Science core collection database from 1980 to 2021, revealing their research hotspots and predicting the future development trends. Over the past 40 years, the number of published papers on abandoned farmland has continuously increased. Research mainly focused on the ecological environment, with natural succession, biodiversity, and vegetation restoration being high-frequency keywords in this field. Research on the social aspects of farmland abandonment has developed rapidly in the past 6 years. Based on these findings, this paper put forward four future research directions: the data source for the extraction of abandoned farmland should transform to high spatial-temporal resolution and hyperspectral remote sensing images; the method should pay more attention to the time series change detection and the application of the model; future research should focus on the economic costs of the reclamation of abandoned farmland and the ecological consequences of such reclamation; and the global ecological impact of vegetation succession after the abandonment of farmland should be further discussed from a broader perspective.

## 1. Introduction

Land use change seriously affects the sustainable development of the global environment [1,2], especially in terms of ecosystem services and biodiversity [3]. Globally, farmland abandonment is one of the major land use changes today [4,5] and a result of the marginalization of farmland driven by social, economic, and environmental factors [6,7]. The main consequences of farmland abandonment are vegetation succession, environmental problems, as well as landscape and socio-economic impacts, with global impacts. Extensive farmland abandonment in one area may lead to large-scale grain imports, often resulting in deforestation [8] in other countries, with effects on ecology, goods, production, and services [9,10]. Recently, farmland abandonment has attracted wide attention from scholars, organizations, research institutions, and the public.

The term “farmland abandonment” has different definitions, depending on the method and content of the study [7]. The Joint Research Centre (JRC) defined farmland abandonment as changes in ecosystem services due to a significant reduction in farmland management. In some studies, farmland abandonment was defined as the cessation of agricultural activity on a given land surface and the subsequent natural restoration of vegetation to grassland [11], shrub vegetation [12], or forest [13], rather than its occupation by other activities (e.g., urbanization or afforestation) [14]. Some studies classified the conversion to forest into a special category of abandonment [15]. At a temporal scale, some studies defined abandoned farmland as farmland left idle for more than 1 year [7]. However, according to the Food and Agriculture Organization (FAO), an idle period of 2 to 5 years must be guaranteed to define farmland as abandoned [16]. Fallows have been assessed as part of a crop rotation cycle to determine whether a plot of land was abandoned or awaiting future use [17]. Some studies distinguished abandoned farmland as a long-term fallow from temporary fallow (defined as farmland that was not cultivated within 1 year) [18].

Previous studies reviewed numerous aspects of research in the field of farmland abandonment. For example, the impact of farmland abandonment on the ecosystem and biodiversity [19] could be regarded as positive or negative [20]. Regarding the positive aspects, farmland abandonment is conducive to the regional biodiversity [21,22,23], increasing the soil organic carbon and vegetation water retention capacity [24,25], and promoting reforestation [26]. However, early succession vegetation after farmland abandonment provides fuel for wildfires [27] and increases the reproductive fitness of weeds, pests, and pathogens on the remaining farmland [28]. Farmland abandonment may also lead to the marginalization of the agricultural cultural landscape, causing the loss of cultural and aesthetic value [29,30]. These effects vary with the geographic area distribution and abandonment period. For example, in Spain, farmland abandonment led to the loss of bird habitat, reduced biodiversity [31], decreased the soil erosion potential within 50 years of farmland abandonment, and increased the incidence of soil erosion within 80–100 years of abandonment [32]. Against the background of food security and the local demand for agricultural products, in some areas, abandoned farmland is being reclaimed. Some examples are the reclamation of some areas after the disintegration of the former Soviet Union [24,33] and the cultivation of formerly abandoned farmland in mountainous areas of China [34,35]. To better understand the ecological consequences and reclamation potential of farmland abandonment, the spatial and temporal patterns and drivers of farmland abandonment distribution have been investigated [36,37,38]. Numerous factors can affect the distribution of abandoned farmland, including natural conditions (such as climate [39], terrain [40], soil [41]), market demand, traffic location conditions, urbanization, and policy [42,43,44].

Generally, literature reviews on farmland abandonment only cover a certain country or region [45,46], thereby lacking a global perspective. Moreover, they largely focus on one aspect of farmland abandonment, failing to outline the current situation or to predict the future development direction. Such an approach limits the ability of researchers to investigate farmland abandonment from different viewpoints and perspectives.

Bibliometrics is an alternative approach to analyze the distribution structure, research topics, and changing trends of a large number of academic publications via mathematical and statistical methods and visualizes the results through visual and intuitive charts [47,48]. Compared with classical review writing, researchers do not need to screen large amounts of literature one by one, which is more convenient, objective, and reliable. The commonly used bibliometric analysis tools include HistCite [49], SATI [50], and CiteSpace [51]. Bibliometrix is an open-source tool developed by Massimo Aria in the R language in 2017. Compared to other bibliometric software packages, such as CITAN [52] and ScientoText, bibliometrix can import and transform data from various database sources, such as the Web of Science, Scopus, Dimensions, and Lens.org, and has more literature information analysis and result visualization functions [53]. Based on the secondary development of bibliometrix, the entire bibliometric steps were assembled into an automated online workflow of biblioshiny. The difference between the two packages is that the bibliometrix’s mode of operation consisted of code commands, whereas biblioshiny uses the Shiny package to package the bibliometrix’s core code and create a web-based online data analysis framework, making it fully available for researchers without any programming skills. It can provide multiple types of statistical methods and a wide variety of visual charts [54].

Bibliometrix has been widely used to quantitatively analyze the literature in geography-related fields. For example, Soler et al. [55] retrieved 1150 publications on rural population reduction, published in the Web of Science and Scopus databases from 1979 to 2018, and used co-word analysis and the co-citation network to conclude that important themes of rural depopulation were relevant to specific geographic areas. Xie et al. [54] conducted data mining and quantitative analysis by retrieving publications in the field of land degradation from 1990 to 2019 from the core collection database of the Web of Science, revealing the research status of global land degradation and evaluating the direction of future research on land degradation. However, publications in the field of farmland abandonment have rarely been reviewed via bibliometrics.

Here, we used the bibliometrix and biblioshiny R language packages to comprehensively analyze the literature in the field of farmland abandonment, considering studies published in the Web of Science core database between 1980 and 2021. First, the development process, current situation, and trend of the research period in the field were identified through the statistical analysis of the annual output of related publications, citations, journal sources, and the main research countries and regions. In addition, collaborative network analysis and clustering of authors, keywords, and topics determined the research hotspots and future frontiers in this field. Based on the above analysis, the problems to be addressed are as follows: (1) What is the trend of article production and citation in the field of farmland abandonment? (2) What are the fluctuation trends regarding the authors, journals, and countries in the field of farmland abandonment? (3) What are the main research directions in the field of farmland abandonment? (4) What is the future research focus in the field of farmland abandonment?

## 2. Data Sources and Methods

### 2.1. Data Sources

**Figure 1:**
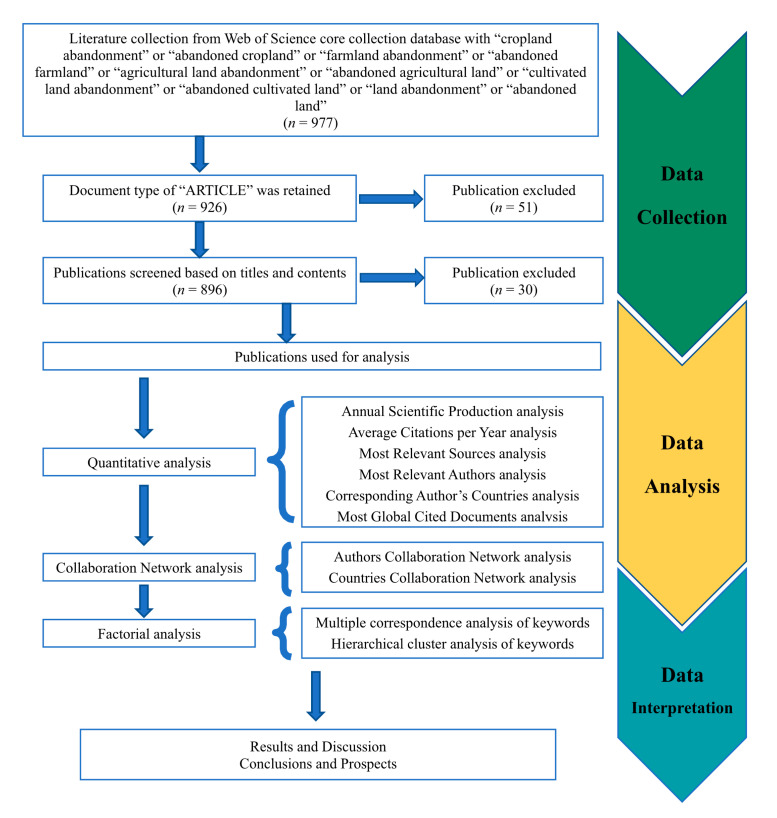
Technical flow of the applied bibliometrics approach.

To improve the representativeness and usability of the data, the data were collected from the Web of Science core collection database [56], which is the largest and one of the most comprehensive bibliometric analysis databases in the world, including data from natural sciences, computer sciences, biology, arts, humanities, and other fields from more than 12,000 high-impact academic journals. By referring to relevant articles in the field of farmland abandonment, we identified the most commonly used keyword “farmland abandonment” [35,57,58,59,60], and the search formula was as follows: T1 = (cropland abandonment) or T2 = (abandoned cropland) or T3 = (farmland abandonment) or T4 = (abandoned farmland) or T5 = (agricultural land abandonment) or T6 = (abandoned agricultural land) or T7 = (cultivated land abandonment) or T8 = (abandoned cultivated land) or T9 = (land abandonment) or T10 = (abandoned land). We limited the publication years to 1980–2021 as some articles will still be published in 2022, whereas the number of articles published before 1980 was extremely small. Overall, 977 publications were found. We focused on journal articles, conference transcript articles, and reviews, excluding comments, news, and letters. After filtering, 926 criteria-compliant publications were downloaded, exported as complete records, and referenced in the format “.txt”. To ensure the completeness and accuracy of the retrieved data, duplicates were manually removed. Finally, a total of 896 publications on farmland abandonment were obtained (Figure 1, Supplementary Table S1). We searched by title rather than by theme, author, abstract, and keywords. In many bibliometric studies, search by title is considered as the most accurate search method [61,62,63], whereas searching by theme/abstract/keywords will produce many false-positive results (the main concern is not the abandonment of farmland itself). Although searching by title can result in the loss of some documents (false-negatives), there will be more false-positive results when searching by theme. In this study, we searched by title and theme, respectively, resulting in 8603 documents being different between the two scenarios. Sorting according to the global citation rate of articles, manually reviewing the titles, abstracts, and keywords of the top 100 articles with the highest citation rates (Supplementary Tables S2 and S3), we found that only 12% of the articles appeared by a theme search focused on farmland abandonment, and most of them also appeared in the articles searched by title. Overall, 42% of the articles on farmland abandonment appeared as marginal research, and 46% of the articles did not investigate farmland abandonment. Among the articles found by the title search, 72% focused on farmland abandonment, 19% only marginally studied farmland abandonment, and 9% did not carry out research on farmland abandonment. Based on this, it was decided to search by title.

### 2.2. Methods

Using the bibliometrix and biblioshiny software packages, the research status and trends in the field of farmland abandonment were reviewed and analyzed. Data analysis included a quantitative analysis of annual scientific production, citations, and journal sources, a collaborative network analysis of authors, countries, and regions, a clustering analysis of document keywords, and the evolution analysis of thematic trends and monitoring methods in abandoned farmland areas during the research period (Figure 1). The present situation and law of research in abandoned farmland were expounded through multiple perspectives to provide a reference and suggestions for future research in this field. The workflow is as follows:

(1) Problems and spans of design research: This paper identified farmland abandonment as the research topic and searched the core database of the Web of Science for articles from 1980 to 2021 by determining search terms, search time, and document types. (2) Data collection: An article database was built [64] by exporting the retrieved documents into the appropriate format of bibliometric tools. (3) Data analysis and visualization: This included co-word analysis [65], collaborator analysis [66], citation analysis [67,68,69], coupling analysis [70,71], and co-citation analysis [72]. For example, two-dimensional maps, dendrograms, and collaborative network diagrams were generated. This step was implemented in the bibliometric software packages bibliometrix and biblioshiny. (4) Data interpretation: the results were analyzed and described, and future research directions in the field of farmland abandonment were proposed.

## 3. Results and Discussion

### 3.1. Analysis of Annual Scientific Results Output and Citation Number

**Figure 2:**
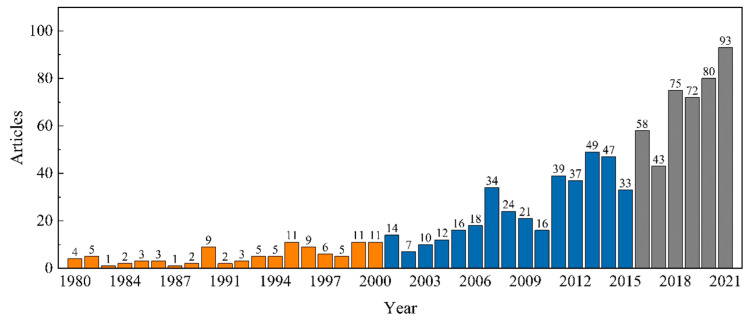
Number of articles on farmland abandonment published from 1980 to 2021 (Note: The different colors in the figure represent the three stages of the number of articles published).

**Figure 3:**
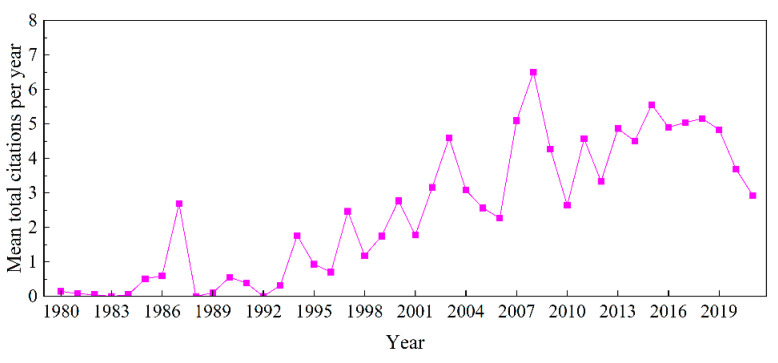
Mean total citations per year on farmland abandonment from 1980 to 2021.

To analyze the general situation and development trend of the research field, the number of published articles (Figure 2) and the mean number of citations (Figure 3) were summarized. From 1980 to 2000, fewer than 11 articles were published in any given year, representing only 10.9% of the total articles published. The mean number of citations can reflect the quality and influence of the article, with four peaks during this period, i.e., in 1987, 1994, 1997, and 2000. The research content was focused on the ecological impacts of farmland abandonment, such as species recovery [73], carbon fixation [74], water retention potential [75], and soil erosion [76]. Immediately after abandonment, because of the lack of vegetation, soil erosion and runoff were serious. In sites that had been abandoned for several years, with a dense vegetation cover, soil erosion and runoff were negligible. There were also studies evaluating the possibility of abandonment reclamation from the ecological perspective [77]. Although in this period studies were scarce, investigating a series of influences after the succession of abandoned farmland from an ecological perspective laid the foundation for subsequent research.

From 2001 to 2015, the number of published articles on farmland abandonment showed an upward trend, albeit with fluctuations, with an average of 25.13 articles per year, accounting for 42.1% of the total published volume. Mean citations peaked in 2003, 2008, and 2015. During this period, sustainability issues, such as climate change and food security, became major concerns. With increasing population size, urbanization, and farmland intensification, the migration of the rural population to cities has led to the large-scale abandonment of farmland. In this period, the contribution of the rational use of abandoned farmland to sustainable development was recognized, and the administration of abandoned farmland has become a hotspot [78,79]. For example, Calcerrada et al. predicted landscape dynamics after farmland abandonment by developing a landscape change transfer matrix model. The authors assumed that, in addition to the ecological value, abandoned farmland can also be used for a series of human activities, such as agriculture, animal husbandry, and tourism [80]. Vegetation succession on abandoned sites and the ecological impacts are some of the main research directions [81,82,83,84]. At the same time, with the emergence of machine learning and different resolution sensors, the use of remote sensing means combined with mathematical models to extract abandoned farmland also attracted wide attention [41,85].

From 2016 to 2021, the number of articles on farmland abandonment increased dramatically, especially in 2020 and 2021, with 80 and 93 articles, respectively. The papers published during this period accounted for 46.99% of the total articles published. In this period, in addition to a change from the traditional ecological perspective to a more modern approach, the research perspective was more diversified, involving the driving factors of farmland abandonment [86,87,88], social, economic, and environmental consequences [6,89], spatial patterns [90], and policy enlightenment [91]. For example, Xu et al. used survey data of rural households in 27 provinces of China to analyze the spatial patterns of abandoned farmland and studied the impact of farmers’ employment on farmland abandonment. Based on the results, labor migration and non-agricultural employment were the main factors driving the abandonment of rural farmland in China [92]. The methods and means of extracting abandoned farmland were also enriched, resulting in a higher accuracy. For example, Yin et al. developed a new method for the extraction of abandoned farmland using a 30-m resolution Landsat satellite time series, with an extraction accuracy of 97% [59]. Although the research content was richer and more diverse, the mean number of citations per project declined after 2018, and the influence of respective articles decreased in 2020 and 2021.

### 3.2. Journal Sources

To study the development of the research area in different journals, the sources of journals were ranked considering the number of publications, the H index, and TC (total citations) (Table 1). The H index was used to measure the importance and impact of the authors’ cumulative research contributions, and the TC represented the total citations for all articles from a journal source. The number of articles published in these journals ranged from 13 to 32. Articles published by *Land Use Policy* over the entire period accounted for 3.6% of the total published articles, followed by those published in *Sustainability*, with 2.9%. In terms of total citations, *Land Use Policy* was the most-cited source, followed by *Catena* with 1642 citations; the ninth-ranked journal, *Remote Sensing*, had the lowest number of citations (165). Taking into account the H index, *Remote Sensing* was again at the bottom of the list, whereas the seventh-ranked journal, *Forest Ecology and Management*, had an H index of 17, which was the same as the first-ranked journal *Land Use Policy*.

**Figure 4:**
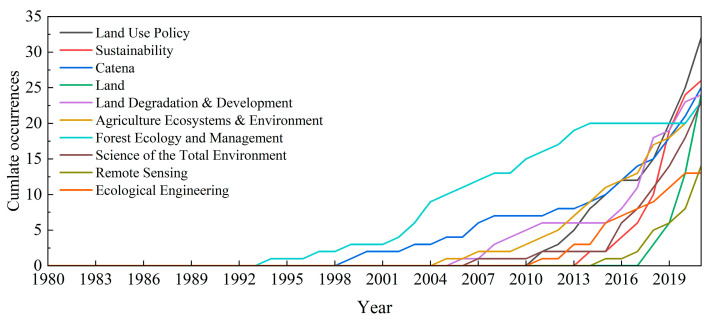
Growth curves of journal sources on farmland abandonment from 1980 to 2021.

According to the journal source dynamics, the evolution curve of the cumulative number of published articles for the 10 most productive journals was plotted over time (Figure 4), obtaining the following information: (1) *Land Use Policy* had the largest production volume and the fastest growth rate during the research period. (2) *Forest Ecology and Management* had a significant production volume before 2014, with a decrease thereafter. (3) *Catena* published the first papers on farmland abandonment in 1999, with stagnation in the middle part of the research period and a significant increase after 2016; *Land Degradation & Development* showed a similar trend. In general, over time, farmland abandonment become the focus of several journals.

### 3.3. Author Analysis

**Figure 5:**
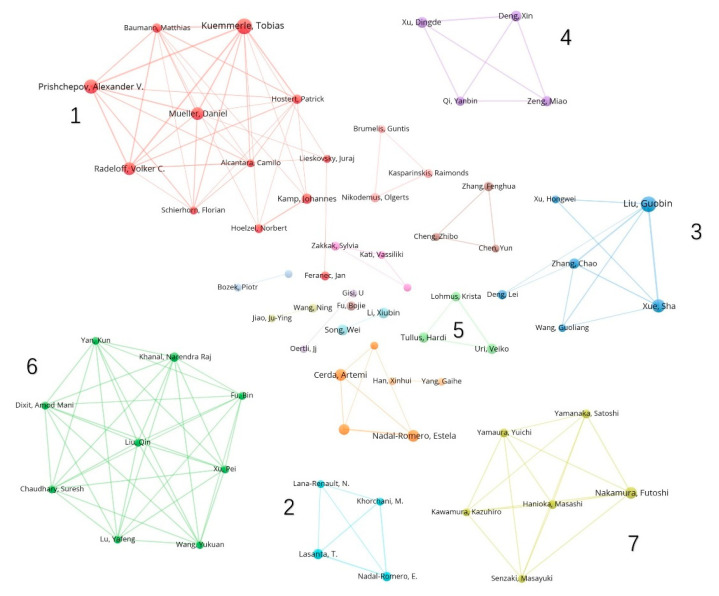
Collaborative network of authors publishing papers on farmland abandonment from 1980 to 2021.

**Figure 6:**
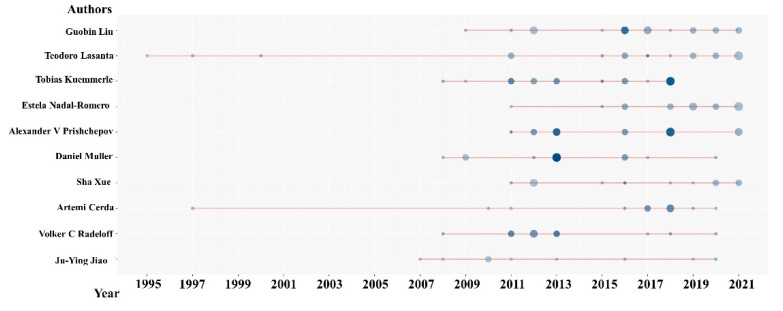
Works of different authors in the field of farmland abandonment over time (Note: N. Articles = number of articles; TC per Year = total citations per year).

We first ranked the most relevant authors in the field of farmland abandonment, along with several bibliometric metrics (Table 2), and subsequently generated the author collaboration network (Figure 5), which reflects the connection of different authors’ knowledge or social levels. This network can be used to identify previously unknown research groups and represents the similarity of research themes [93]. Finally, we plotted the authors’ changing results over time (Figure 6). Articles fractionalized (AF) indicated the contribution rate of the author to the article in the article jointly published by the author and other authors. Overall, 2747 authors contributed to publications in the field of farmland abandonment. Among them, the top three authors were Guobin Liu, Teodoro Lasanta, and Tobias Kuemmerle, with 19, 18, and 16 published papers, respectively (Table 2). Using the H index to measure the importance and influence of the cumulative research contribution, the author with the highest H index was the German scholar Tobias Kuemmerle, with a H index of 15 and a total citation number of 1813, indicating the high quality and influence of his published papers.

The results in the Author Collaborative Network (Figure 5) were divided into 13 clusters, with each color representing a group of collaborative authors. The larger the circle, the more productive the author. The most representative seven groups of authors were selected, and their studies were analyzed. 

The first group examined farmland abandonment in eastern Europe and the former Soviet Union after the disintegration of socialism. Different land policies, economic development, and reform strategies led to large differences in the abandonment rates among different countries [94,95]. During the transition period of 1990–2000, the agricultural sector in Russia, Latvia, and Lithuania declined sharply, while the socio-economic and agricultural infrastructure in Lithuania were more suitable for agriculture, leading to a low abandonment rate. In this period, the Russian government almost completely abolished agricultural subsidies, which led to a high abandonment rate [96]. In contrast, the Belarusian government provided more support for agriculture, leading to a low abandonment rate. Poland and Slovakia, located near the edge of the Carpathian Mountains, are largely affected by terrain and market access, which resulted in a high abandonment rate. However, due to the adoption of private agriculture in the socialist period in Poland’s plain area, the agricultural sector in this area quickly adapted to the post-socialist framework, which resulted in a low abandonment rate [94].

The second author group mainly studied the effects of farmland abandonment on soil and hydrological conditions in the Mediterranean region [97,98]. For example, in the southeast of Spain, soil degradation after farmland abandonment is serious. Droughts led to the generation of a surface crust, which reduces the permeability and increases surface runoff and soil erosion [76]. In the Pyrenees Mountains, farmland abandonment has greatly increased the contents of suspended sediment in the water, which has affected the water supply and agricultural activities in the downstream area. However, the local environment is humid, which is conducive to afforestation. Vegetation has increased the soil stability in this area, resulting in reduced soil erosion, surface runoff, and flood probability [5,97,98].

The third group of authors focused on the effects of farmland abandonment on the Loess Plateau of China, such as the effects on soil carbon sequestration [99] and the microbial community [100]. For example, Zhang et al. reported that the vegetation coverage rate decreased in the 10 years before abandonment and returned to normal levels after 15–20 years of abandonment [101]. Deng et al. studied the organic carbon reserves of soil after abandonment on the Loess Plateau, which decreased significantly in the first 20 years and recovered to pre-abandoned levels 30 years later [102]. Similar to the second group, this author group studied the impacts of farmland abandonment on the land. However, whilst the second group focused on soil properties and runoff, this group mainly investigated the succession of vegetation communities and organic carbon reserves over time.

The fourth group of authors mainly analyzed the factors driving and influencing the abandonment of rural farmland in China. In this country, more areas are abandoned in mountainous and hilly areas than in the plains [101]. Xu et al. revealed a significant inverted U-shaped relationship between farmers’ employment outside the agricultural sector and farmland abandonment; based on their results, non-agricultural labor does not necessarily lead to farmland abandonment. On the one hand, when rural laborers are employed in non-agricultural sectors, this leads to a shortage of the agricultural labor force, a lack of the land transfer market in rural areas, and insufficient labor resources for families to maintain the production of their existing land, often resulting in extensive farmland abandonment. On the other hand, most farmers are unable to integrate into urban areas and are unwilling to sacrifice their land; they may, therefore, choose outsourcing to avoid giving up their farmland [92,103]. Another study shows that the internet can help farmers significantly reduce farmland abandonment [104].

Similar to the second and third groups, the fifth group of authors also discussed the relationships between farmland abandonment and vegetation and species diversity but paying more attention to the afforestation on abandoned farmland in eastern and northern Europe. For example, hybrid aspen plantations planted on abandoned farmland in Estonia were monitored, and their impacts on understory vegetation and species numbers were discussed [105], as well as the effect of alder on soil nitrogen [106]. In another study, the aboveground biomass, belowground biomass, and nutrients of natural birch forests on abandoned farmland in Estonia were studied [107].

The sixth group of authors focused on abandoned farmland in the mountainous areas of Nepal, such as the Himalayan mountains [108,109] and the Dordi River basin [110]. For example, Chaudhary et al. evaluated the temporal and spatial degradation of abandoned farmland in the Dordi River basin and analyzed its causes and the resulting ecological environment risks. Of the total farmland surveyed, 92% had been completely destroyed, leading to landslides, debris flow, rock fall, gully formation, soil erosion, and limestone pits, thus increasing the negative impact on land resources and vegetation succession [109]. The authors also evaluated the temporal and spatial distribution and driving factors of farmland abandonment in the mountainous areas of Nepal and discussed the ecological and social impacts of abandonment. Based on their results, farmland abandonment is widespread in the hills and mountains of Nepal, with one fifth of the total farmland area being abandoned from 2001 to 2010. Population growth, migration, urbanization, inconvenient transportation, scattered living, poverty, and lack of land policies are the main factors driving farmland abandonment in Nepal. In turn, abandoned farmland restricts the supporting structure of terraced fields in this area, causing natural disasters, such as landslides and mud rock flows, and impeding natural succession. Overall, the results on the ecological environment and the rural society are negative [108,111].

The seventh group of authors studied the changes in species distributions after farmland abandonment, focusing on birds. The main study area was Hokkaido, Japan, where abandoned farmland and wetlands coexist. The authors assessed the landscape patterns of the abandoned farmland [112] and compared the bird biodiversity of the abandoned farmland with that of other local land use types [113,114], indicating that the bird abundance in abandoned areas is equivalent to that of wetlands. Different from the European region, the positive impact of farmland abandonment in this region is greater than the negative impact. Abandoned farmland may evolve into wetlands and forests, which can provide habitat and recovery opportunities for local species. In this context, the management of abandoned farmland in this area is crucial to the protection of bird species in the agricultural landscape.

At least five of these clusters were interested in ecological issues, whereas the remaining two clusters reported social concerns. Based on different natural conditions, policies, and systems, studies on farmland abandonment showed obvious regional differences.

Considering the authors’ production over time (Figure 6), Teodoro Lasanta and Artemi Cerda were the two authors with the longest-standing involvement in research on farmland abandonment. Daniel Muller published four articles with other authors in 2013, with the highest annual total citation number of 68.6. Abandoned farmland in Europe was extracted mainly by means of remote sensing methods. For example, the abandoned farmland [115] in 30 countries, including central and eastern Europe, was quantified by support vector machine classification in an article in cooperation with Alcantara et al., using MODIS NDVI time series satellite images. A spatial allocation model that obtained a time series of abandoned farmland [24] in Russia, Ukraine, and Belarus from 1990 to 2009 was developed in cooperation with Schierhorn et al. Four articles published by Tobias Kuemmerle in collaboration with other authors received 58.2 annual total citations, also studying farmland abandonment in Europe [116,117]. Two authors, Daniel Muller and Tobias Kuemmerle, belong to the first cluster of the author collaboration network (Figure 5), with most of the total citations.

### 3.4. Analysis of the Distribution Characteristics of the Main Research Countries/Regions

**Figure 7:**
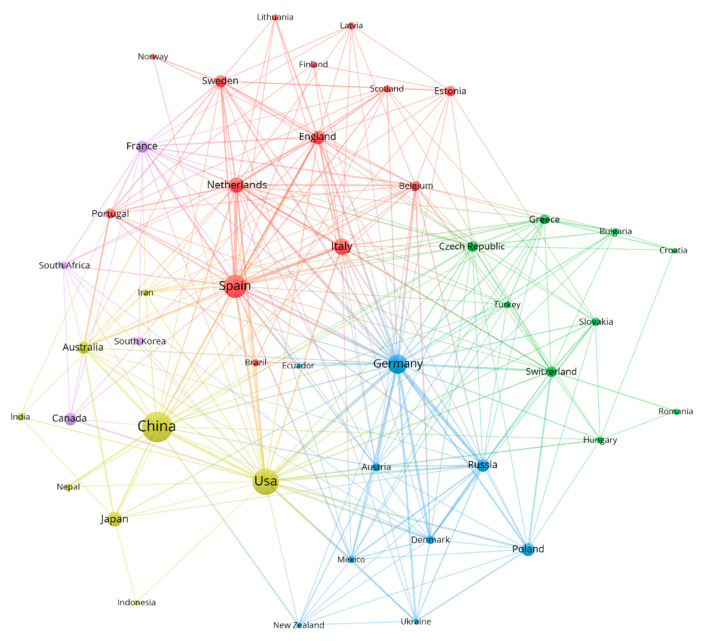
National collaborative network for research on farmland abandonment from 1980 to 2021.

To identify the key countries and regions in terms of publication number and impact, the cooperation network between countries and regions was analyzed (Figure 7). Between 1980 and 2021, a total of 62 countries or regions published papers. Among the top 20 countries regarding publication number (Table 3), there were three Asian countries (China, Japan, Korea), two American countries (the United States, Canada), 14 European countries (Spain, Italy, Germany, Poland, the Netherlands, the United Kingdom, France, Estonia, Sweden, Switzerland, Russia, Greece, Slovakia, Portugal), and one Oceanian country (Australia). Among the top five countries with the largest numbers of publications, three were European countries. Based on our findings, European authors were more concerned about the issue of farmland abandonment. China was the only developing country among the top five countries regarding publication number, ranking first and having published 193 papers. However, the mean number of citations per article was low, with only 20.71, indicating that Chinese authors need to pay more attention to the quality of their articles. The countries with the highest mean citation numbers were the Netherlands, followed by Switzerland, with 96.88 and 95.07, respectively, but with fewer publications, ranking ninth and 15th, respectively.

**Figure A1:**
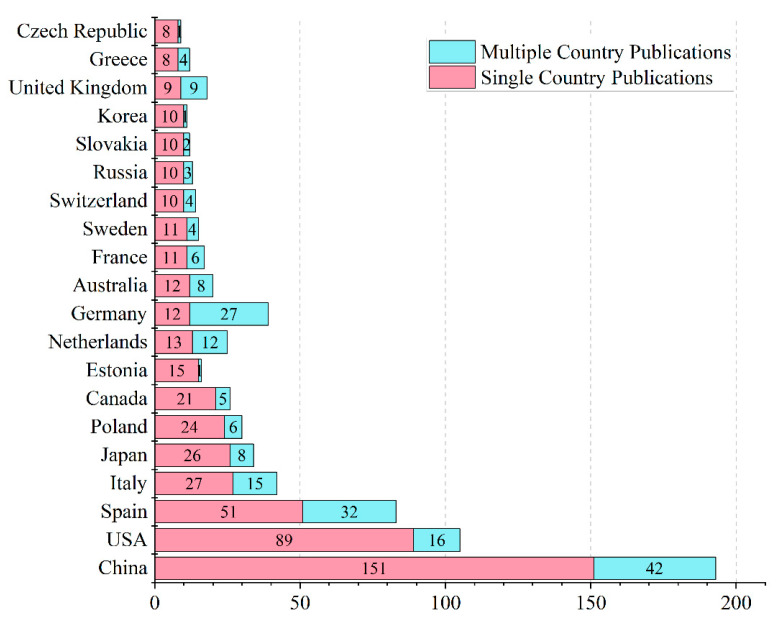
Number of independent and cooperative publications in the 20 countries with the most published articles from 1980 to 2021.

Based on the national collaboration network (Figure 7), the most closely cooperating countries were Germany and the United States, Russia and Germany, and Spain and the Netherlands, with 17, 15, and 19 cooperation articles, respectively (the thicker the lines, the closer the connection between countries). The node size represents the number of articles published in cooperation with other countries. The countries with the most co-published articles were China, Spain, and Germany, with 42, 32, and 27 articles, respectively (Figure A1), accounting for 16%, 12%, and 10% of the total output of co-published articles, respectively. China and Spain published a larger number of articles independently, with Germany, the UK, and the Netherlands focusing on collaborative research with other countries.

### 3.5. Document Analysis

#### 3.5.1. Analysis of Highly Cited Papers

The top 10 cited articles (Table 4) reflected the most influential content in the field of farmland abandonment from the perspective of citations, including seven research papers and three reviews. The article with the highest overall number of citations proposed that determining the ecological restoration goal of abandoned farmland is a challenge that people will face in the future. It was also proposed that the future research direction should be combined with land use policy [78]. Gellrich et al. [118] developed an economic model to analyze the driving factors of natural forest regeneration in the Swiss mountains, showing that natural forest regeneration is more likely to occur in areas where farmland has been abandoned. Similar findings were found in other mountainous areas, such as the Alps as well as in Sweden, Poland, Denmark, the Baltic Sea, and parts of Slovenia. Other studies focused on Europe; for example, the 10th article [119] used the revised CAPRI model to assess the consequences of farmland abandonment in the EU under the Common Agricultural Policy (CAP). In the whole of the EU, the distribution of abandoned farmland in different countries, regions, and farm types is uneven. Abandoned farmland caused by agricultural and trade policy reforms may have a significant impact on rural livelihoods and the environment, such as a reduced biodiversity.

In addition, the paper ranked No. 2 [120] used the Dyna CLUE model to conduct a clear simulation and spatial evaluation on the natural succession track of abandoned farmland and vegetation in Europe from 2000 to 2030. The simulation results show that abandoned farmland in Europe mainly occurs in the mountainous regions. The natural succession after abandonment mainly depends on local conditions, such as climate, population density, and terrain [81,121,122,123]. The seventh-ranked article assessed the hydrological impacts of land abandonment in the Mediterranean region of Europe, focusing on water resource availability and soil erosion [45]. This study shows that vegetation succession after abandonment is related to many factors, including soil depth and fertility, slope aspect, and climate (annual average precipitation, evapotranspiration), among others [124]. In the Mediterranean region, abandoned farmland is mainly concentrated in mountain terraces and semi-arid areas, such as southern France, southeast Spain, south central Portugal, and Italy [81,125,126]. In terraced areas, such as the Iberian Mountains and the Pyrenees Mountains in Spain [84,127,128], farmland abandonment has resulted in landscape degradation and soil erosion, sediment deposition in river channels, and increased surface runoff. In semi-arid areas, higher temperatures, less precipitation, and long-term drought impede vegetation succession on abandoned farmland. The resulting surface crust reduces the infiltration rate, increases surface runoff, and leads to surface erosion [129]. On the contrary, in humid areas, the succession of vegetation dominated by herbaceous and shrub species is rapid [120].

After the collapse of the socialist system in eastern Europe and the former Soviet Union, under the dual impact of the system and the economy, farmland abandonment differed among different European countries. The article ranked No. 8 [42] studied the spatial distribution and driving factors of farmland abandonment in western Ukraine after the collapse of the socialist system, identifying differences with other parts of Europe. In western Europe, abandonment is mainly driven by industrialization, market orientation, and urbanization, such as in areas around the Alps and Romania [130]. The abandonment rate in the plains of western Ukraine is high, whereas that in the marginal areas is low. In the transitional period, the agriculture in this area was not yet industrialized and dominated by traditional self-supporting agriculture. The plain soil is relatively barren and not suitable for farming, and the reduction of the agricultural labor force due to large-scale migration and regular remittances from family members seeking non-agricultural employment in other regions can maintain the livelihood of the farmers. In addition, compared with other eastern European countries, such as Latvia, Poland, Russia, Lithuania, and Estonia [91,131,132], the abandonment mode in western Ukraine is different, most likely because of differences in socialist land ownership patterns, post-socialist land reform strategies, and rural population densities [94,115]. With this in mind, the factors driving farmland abandonment cannot be generalized among countries that are also impacted by the collapse of socialism.

**Table 4:** Highly cited articles on farmland abandonment from 1980 to 2021 (Note: TC per Year = total citations per year; LCR = local citation rate).

Paper	Year	Total Citations	TC per Year	LCR	Source
What’s new about old fields? Land abandonment and ecosystem assembly [78]	2008	531	35.4	12.81	*Trends in Ecology and Evolution*
Combining top-down and bottom-up dynamics in land use modeling: exploring the future of abandoned farmlands in Europe with the Dyna-CLUE model [120]	2009	449	32.07	8.02	*Landscape Ecology*
Tree line shifts in the Swiss Alps: Climate change or land abandonment? [133]	2007	433	27.06	0	*Journal of Vegetation Science*
The global potential of bioenergy on abandoned agriculture lands [79]	2008	418	27.87	9.09	*Environmental Science & Technology*
The potential for carbon sequestration through reforestation of abandoned tropical agricultural and pasture lands [74]	2000	347	15.09	2.88	*Restoration Ecology*
Agricultural land abandonment and natural forest re-growth in the Swiss mountains: A spatially explicit economic analysis [118]	2007	342	21.38	23.98	*Agriculture Ecosystems & Environment*
Hydrological and erosive consequences of farmland abandonment in Europe, with special reference to the Mediterranean region—A review [45]	2011	288	24	17.71	*Agriculture Ecosystems & Environment*
Patterns and drivers of post-socialist farmland abandonment in Western Ukraine [42]	2011	287	23.92	27.87	*Land Use Policy*
Farmland abandonment: threat or opportunity for biodiversity conservation? A global review [19]	2014	262	29.11	21.76	*Frontiers in Ecology and the Environment*
Policy reform and agricultural land abandonment in the EU [119]	2013	259	25.9	21.24	*Land Use Policy*

The third-ranked article mainly studied the driving mechanism of grassland abandonment on forest expansion. Although the total citation number was high, the local citation rate (LCR) was 0, indicating that the article was not correlated to the field of farmland abandonment. The fourth-ranked article estimated the abandoned global farmland area via land use data from the global Environment 3.0 historical database and MODIS satellite images, demonstrating the bioenergy potential of the global abandoned farmland [79] using the CASA ecosystem model. Another article reviewed farmland abandonment and its impact on biodiversity from a global perspective by investigating studies from different countries, ranking No. 9 [19]. Reviewing 276 papers, this paper found that the impact of abandoned farmland on biodiversity differs regionally. Whilst most studies in Europe reported a negative impact of farmland abandonment on biodiversity, studies in central and southern America largely found positive impacts on biodiversity. In the latter region, agricultural expansion is the primary issue in biodiversity conservation. Most studies in North America discussed the processes and mechanisms that lead to or accompany farmland abandonment, largely neglecting its impact on biodiversity. Through further analysis, the authors found that the differences were mainly related to the environment, the methods used by researchers, and the concerns about the landscape before and after abandonment.

#### 3.5.2. Keyword Analysis

By comparing the top 20 author keywords (DE) and keywords-plus (ID) most related to the field of farmland abandonment (Table A1), farmland abandonment/agricultural land abandonment, succession, soil erosion, biodiversity, restoration, and Europe were the six keywords shared by the two. Different author keywords clearly defined the content, approach, or method of the author’s research, and the common ones were farmland abandonment, land use change, land use, secondary succession, and afforestation. The keywords-plus in the Web of Science database could objectively describe the content of the article from a macro-perspective, such as farmland abandonment, vegetation, dynamics (mostly referred to soil dynamics), forest, and management. This indicates that, currently, studies focus on the natural succession of abandoned farmland and the impacts of human activities on the ecological environment.

#### 3.5.3. High-Frequency Keyword Cluster Analysis and Multiple Correspondence Analysis

**Figure 8:**
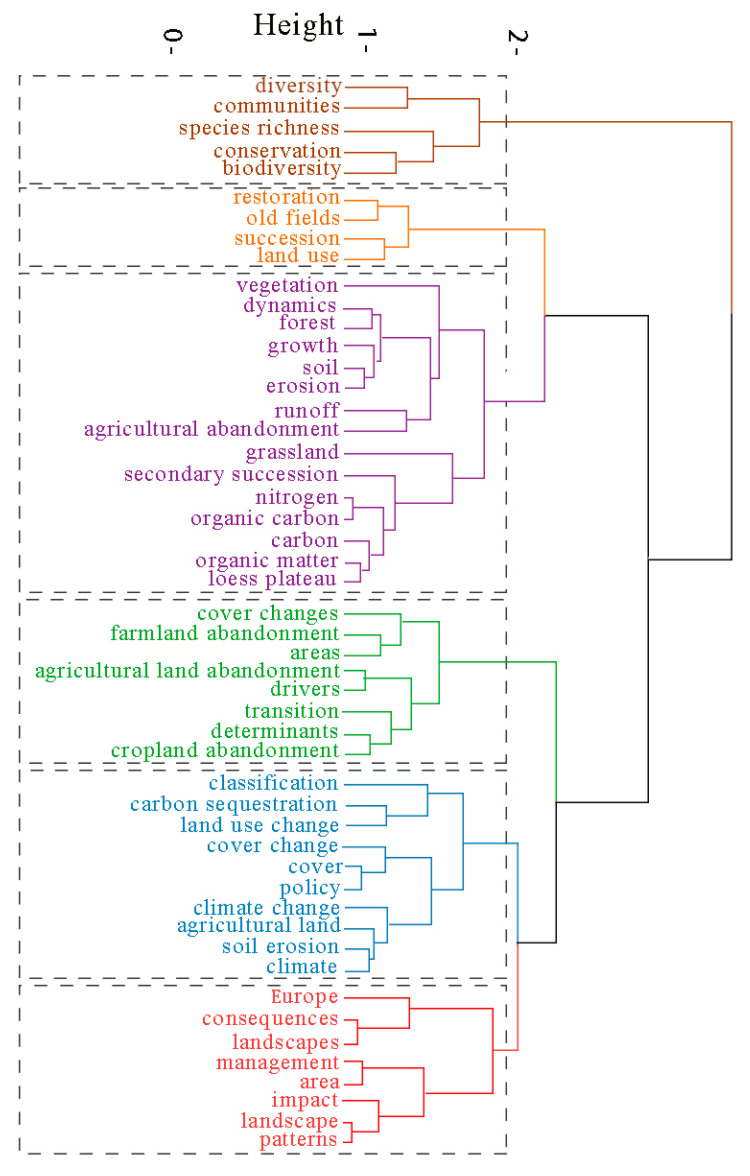
Hierarchical clustering analysis of keywords in the field of farmland abandonment.

**Figure A2:**
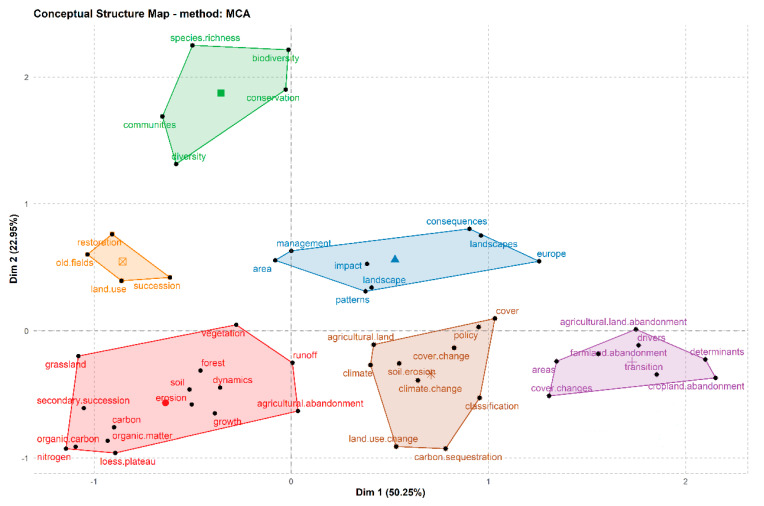
Multiple correspondence analysis of keywords in the field of farmland abandonment.

Using the functions of cluster analysis and multiple correspondence analysis, biblioshiny could reflect the hotspots, themes, and writing directions in the field of farmland abandonment. Cluster analysis was used to cluster complex keyword–network relationships into several relatively simple groups [134]. The keywords with the highest similarity were merged into one cluster, and the cluster with the highest similarity was merged into a large cluster, and so on, until all categories were finally merged into one class, forming a dendrogram representing the close or alienation relationship of the keywords in the field of farmland abandonment (Figure 8). Multiple correspondence analysis (MCA) resulted in the formation of an intuitive two-dimensional graph by reducing multi-dimensional data to low dimensions, using a plane distance to reflect the similarity among keywords. A close proximity to the center indicates that this type of topic had received higher attention, whereas a greater distance of keywords from the center indicates less attention or a greater degree of deviation from the topic [135] (Figure A2).

The first cluster category focuses on the study of biodiversity, species richness, and plant communities after farmland abandonment. There are obvious regional differences in the ecological and environmental consequences of farmland abandonment. For example, most studies in European regions show that the encroachment of vegetation on bare land and original farmland after abandonment will have a negative impact on lizards, birds, and other animal species [136]. One study in the Mediterranean Sea shows that vegetation encroachment after farmland abandonment will cause the loss of bird habitats. Regular burning, grazing, and other means may help to protect bird diversity. However, a study on Hokkaido, Japan, obtained opposite results. Most wetlands in this area have been transformed into farmland, which is now abandoned. The authors assume that the abandoned farmland can be used as a habitat for wetland birds through management, helping to promote biodiversity [137,138]. In southeast Europe, such as in Bulgaria and Croatia, plant species diversity is greatly affected by elevation and temperature. In areas with low elevation and cold temperatures, species are more abundant [139]. However, the richness of bird species declines after farmland abandonment, highlighting the importance of the management and maintenance of traditional rural landscapes in this area.

The second cluster category mainly contains studies on vegetation and species restoration over time after farmland abandonment. Zhang et al. [140] studied the vegetation community and soil characteristics of abandoned farmland in the Qinling Mountains of China after a period of natural succession, indicating that natural succession is conducive to the ecological recovery of abandoned farmland, which is characterized by high biodiversity, high soil fertility, and high species richness. The species richness of the community increased in the first 15 years after abandonment [100,102,141]. However, Yannelli et al. [142] reported that, in arid regions, natural succession may not be the best method for ecological restoration, and soil erosion may occur during succession, preventing the establishment of a vegetation cover. Restoration should be adapted to local conditions, taking into account environmental factors such as climate and topography in different regions. Standish et al. [143] also considered the impact of alien species invasion on the restoration of abandoned farmland vegetation, indicating that, during natural succession, alien species are more competitive than native ones. The authors also highlighted that restoration should be more focused on maintaining ecosystem services at the site, rather than attempting to restore historic ecosystem states.

The third category contains publications on the impacts of natural succession on forests, soils, and microbial communities, mostly in the mountainous areas of Europe and on the Loess Plateau of China. Cojzer et al. [144], in Slovenia, compared the species structure, quantity, and tending time of young forests formed by secondary succession and artificial reforestation after farmland abandonment. The results showed that the young forests formed by secondary succession had great structural complexity and biodiversity, and the management of these stands was more conducive to local ecological restoration than reforestation. Treml et al. [145] evaluated the changes in tree coverage and number in the Sudeten Mountains of Central Europe through aerial images; based on their results, farmland abandonment is the main cause of forest intensification. Chinese authors studied the vegetation succession and soil carbon reserves after farmland abandonment on the Loess Plateau, reporting that vegetation coverage, species richness, and soil carbon reserves significantly increased over time, recovering to the original levels [146] within about 15 years, increasing significantly within 15 to 25 years; however, after 50 years, they were still lower than those of natural grassland [147,148]. In one study, the introduction of alien species reduced the time to restore vegetation to its original level to 11 years [149]. Another study showed that the reforestation of farmland after abandonment can significantly increase soil fertility [150].

The fourth category focused on methods to extract abandoned farmland, studying the mode, driving force, and spatial determinants of abandoned farmland. The commonly used method identified abandoned farmland by analyzing the inter-annual land use change in a given study area. Subsequently, the driving factors and spatial determinants of farmland abandonment [151,152] were determined by mathematical models. For example, Baumann et al. [42] plotted the abandoned farmland in the post-socialist period of western Ukraine, using the support vector machine classification method and Landsat images from 1986 to 2008, and determined the spatial determinants of abandonment using a combination of the optimal subset linear regression model and hierarchical division. Based on the results, 30% of the farmland was abandoned in the post-socialist period, mainly as the result of the combined impact of the system and the economy. A study in southern Chile [153] extracted the abandoned farmland by comparing land use in 1985 and 2007, using a spatial explicit statistical model. The authors showed that soil quality, distance to secondary roads, and agricultural subsidies were important drivers of local farmland abandonment. In eastern Poland, Zgłobicki et al. [154] showed that natural conditions (topography, soil), socioeconomic characteristics (farmland area, forest cover changes, farm size), and agricultural policies were the largest drivers of local farmland abandonment.

The fifth category was similar to the third and fourth categories. However, this category not only constructed the extraction method of farmland abandonment but also focused on the bioenergy potential abandoned farmland [155], estimating that the potential is 8% of the global demand for primary energy [79]. Schierhorn et al. developed a model for mapping abandoned farmland and calculating carbon sinks in such farmland, based on acreage statistics. The authors provided the means to map abandoned farmland in areas in which remote sensing is difficult to perform and stated that the rich carbon sinks of abandoned farmland can mitigate global climate change; reclamation of such areas is likely to lead to large amounts of carbon emission [24].

In Europe, with the highest number of publications on farmland abandonment, studies on the distribution, patterns, consequences, and impacts of farmland abandonment formed a separate cluster. The causes of farmland abandonment in Europe are various, depending on the region and period under consideration. Due to natural conditions, historical development, and economic, social, and demographic backgrounds, agricultural conditions vary from region to region. For example, Terres et al. established a unified indicator of the driving factors of abandonment through a comprehensive analysis of farmland abandonment in different regions of Europe, including the aging population, the low population density, the low farm income, the small scale, and the poor implementation of agricultural plans. This indicator covers the possible drivers of abandonment in all EU countries and can be flexibly applied according to the background of different regions [156,157,158]. Prishchepov et al. [132] discussed the impacts of institutional changes in different countries in eastern Europe and the former Soviet Union on farmland abandonment after the collapse of socialism. The authors stated that the reform of systems and policies is an important factor indirectly driving farmland abandonment in this region. In places where the institutional change of agricultural land management is relatively small, and where the new institutions after the institutional change are relatively strong, the abandonment rate is the lowest. On the contrary, countries that delay the formulation of new agricultural production systems and regulations have a higher abandonment rate. In addition, the European region also focuses on the impact of farmland abandonment on ecology. For example, in the Alps, farmland abandonment leads to spontaneous reforestation, reducing grassland species and negatively impacting plant diversity [159]. Another study assessed the carbon storage dynamics of abandoned farmland in the former Soviet Union and showed that the abandoned farmland is transformed into an ecosystem dominated by grassland, resulting in an increase in carbon sequestration and a significant impact on the carbon sink in the region [160].

#### 3.5.4. Analysis of the Research Theme Evolution

**Figure A3:**
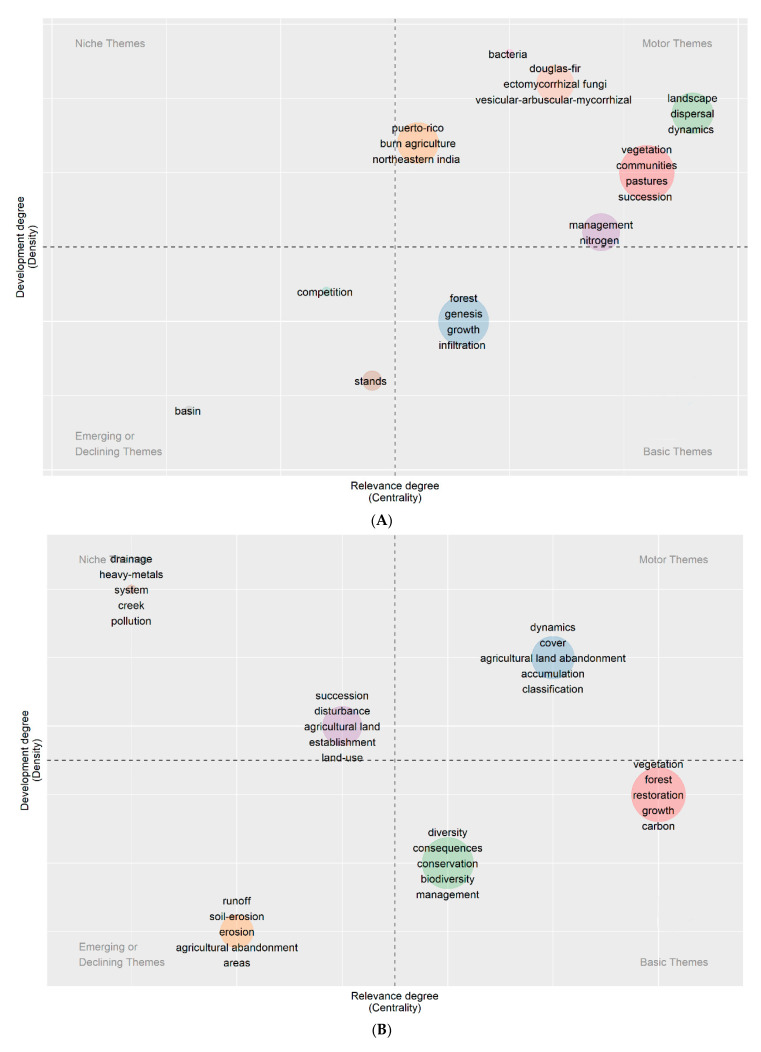

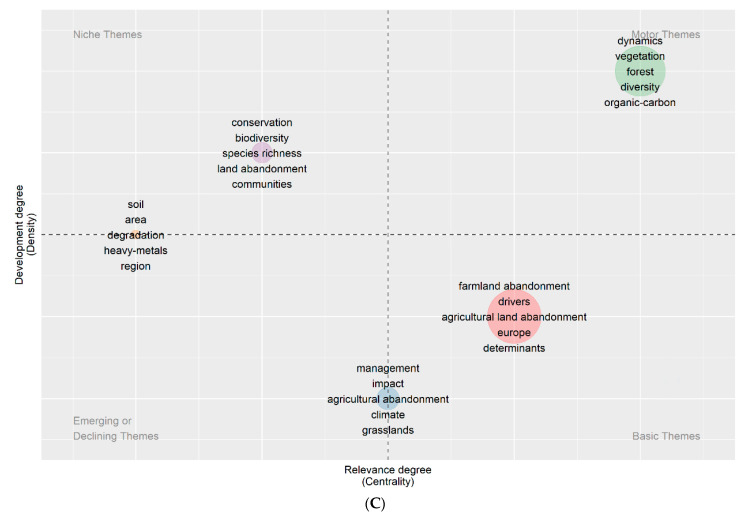
Strategic coordinates of the farmland abandonment period 1980–2000 (**A**), 2001–2015 (**B**), and 2016–2021 (**C**).

Theme evolution analysis can explore the changing laws, relationships, paths, and trends of the content, and intensity and structure of the research topic over time. Here, the study period was divided into three stages, with strategic coordinates being drawn (Figure A3). Overall, the research topics in the field of farmland abandonment experienced a process from increase to decrease to increase. In the initial stage of 1980–2000 (Figure A3), the core themes with high maturity were vegetation, landscape, dynamic degree, and management (the first quadrant in Figure A3). For example, a Swiss study provided advice on the local agricultural management policy [161] by interviewing people regarding their preferences for reforestation after farmland abandonment. However, Puerto Rico was the area of concern for this phase, mainly because it was funded by the US National Fund at the time [74] and because of the support of the University of Puerto Rico in this field [162]. The themes competition and basin disappeared in the following stage. In addition, the natural succession of abandoned farmland resulted in the formation of new forests after the abandonment, and forest communities, soil, and vegetation became research hotspots.

During the development stage from 2001 to 2015 (Figure A3), the theme type with high maturity was relatively single, and the dynamic degree (forests dynamic, land cover dynamics, vegetation dynamics) was still the core theme of this period. The new core themes, such as land cover and agricultural abandonment, were consistent with the study of land cover changes [81] following agricultural abandonment in the Mediterranean mountains and the role [80] of farmland abandonment in landscape dynamics in central Spain, using three Landsat land cover maps. Runoff and soil erosion were emerging themes in this period. However, the occurrence in the third quadrant showed that the research on this theme was not mature enough, and the topics such as vegetation, forest, and diversity are still hot topics for future research, getting closer to the first quadrant. Notably, the top 10 most-cited articles (Table 4) were all published in the second half of this period.

During the active phase of 2016–2021 (Figure A3), studies on forest, vegetation, dynamic degree, and biodiversity formed the core of the field of farmland abandonment research. Europe, as an important area in respective research, is distributed in the fourth quadrant as a keyword with a high number of occurrences. Along with the analysis of the driving factors and the determinants of abandonment, this field may become a research hotspot in the future. Whereas previous studies on farmland abandonment in Europe were mainly conducted in typical European countries and regions, with a small scale, with the increase in achievements and the improvement of technical means, large-scale studies covering the entire European continent appeared in this stage. For example, Walter et al. evaluated the causes and consequences of farmland abandonment in Europe by summarizing previous studies [6], whereas Ustaoglu et al. evaluated the drivers of farmland abandonment and their sustainability impact on society and the environment [86]. Lasanta studied the spatial and temporal processes and drivers of farmland abandonment in Europe [87]. Research on abandoned farmland in typical regions such as Ukraine [163], Russia, and Kazakhstan [37] has also been revitalized. Compared with previous stages, this stage of research pays more attention to the determinants of the reclamation of abandoned farmland, with the aim to avoid the ecological impacts of farmland expansion.

#### 3.5.5. Analysis of the Monitoring Method Evolution

This study reviewed the abandoned farmland monitoring methods used in the database. The representative methods in this field changed over time (Table 5) and can be divided into three types: (1) Field survey; (2) remote sensing image classification methods, such as visual interpretation, supervised classification, object-oriented classification; and (3) change detection methods, including vegetation index change detection and multi-temporal remote sensing image time series change detection. At present, the monitoring of abandoned farmland mainly considers multi-source remote sensing images and the integration of multiple monitoring methods. Over time, the monitoring methods changed from field survey and the visual interpretation of aerial photos to remote sensing. Remote sensing data also changed from small-scale- and medium low-spatial-resolution data to large-scale- and high-spatial-resolution data [94,116,131,164,165].

(1) Field survey. The largest advantage of this method is that it can explain the mechanism behind farmland abandonment, although it is difficult to obtain a complete view of all abandoned farmland areas in a given region due to the lack of spatial details. Therefore, the extraction of abandoned farmland often uses field survey data coupled with other models. For example, Florian et al. distributed the survey data of European Russia, Ukraine, and Belarus to the remote sensing image data through the spatial allocation model, obtaining an accuracy of 65% [117]. Raymond et al. combined geographic data obtained from field surveys with aerial photos to extract abandoned farmland in southern France, which accounted for 5% of the study area [166].

(2) Classification of remote sensing images. Visual interpretation methods are mostly applied to Mediterranean mountains [41,42,81], and the data sources are mostly aerial photos. Visual interpretation has the advantages of simple operation and high accuracy, although it is greatly influenced by the interpreter, and the degree of automation is low. It requires considerable manpower and time to process data, and the data are difficult to obtain. The application of supervised classification originated in eastern Europe and the former Soviet Union [96,121,132,167], generally applying Landsat TM/ETM+ data. Supervised classification can use prior knowledge to improve classification accuracy by learning sample features. This method can be applied to high-, medium-, and low-spatial-resolution remote sensing data at the same time, allowing its wide use in abandoned farmland monitoring. The disadvantage is that the sample selection is highly subjective, and it is difficult to distinguish land types with similar spectral characteristics.

(3) Change detection method. Some authors took more than 30 countries in central and eastern Europe as the research area and used MODIS NDVI time series products to extract abandoned farmland. This product has a high time resolution, and the characteristics of abandoned farmland can be compared with the growth cycle characteristics of vegetation types. However, the spatial resolution is low (250 m). This study also combines SVM classification, obtaining an accuracy of 50.7% [115]. Estel et al. used MODIS NDVI time series products combined with random forest classification to monitor abandoned farmland throughout Europe and mapped the reclamation range of farmland, with an accuracy of 90.1%. Farmland was mainly abandoned in eastern Europe, southern Scandinavia, and the European mountains. Reclamation is also common, mainly in eastern Europe (such as Russia in Europe, Poland, Belarus, Ukraine, and Lithuania) and the Balkans [85]. Vegetation index change detection can identify the growth difference between crops and natural vegetation, with low data redundancy and high method fault tolerance, but, due to the influence of image spatial resolution, the recognition accuracy of abandoned farmland is low [79,115]. In recent years, the most commonly used method was Landsat remote sensing image time series change detection. Its key is the initial classification accuracy of the image, and the change detection accuracy is accumulated by different time phase classification accuracies. Yin et al. used Landsat images from 1985 to 2015 and the LandTrendr model to detect changes in the time series of multi-temporal remote sensing images, applying the object-oriented classification method. This method is not only based on the spectral information and texture information of abandoned farmland but also considers the geometric information. By formulating a variety of rules to constrain the target land class, it has significant advantages in improving the initial classification accuracy of remote sensing images. The overall accuracy of the study on the classification of abandoned farmland is 97%, which is better than that obtained using the pixel level change detection (82%) [59].

## 4. Conclusions and Prospects

We conducted a quantitative analysis and visualization of articles published in the field of farmland abandonment from 1980–2021 at the macro-scale by using the bibliometrix and biblioshiny software packages, based on the Web of Science core collection database. From this, we determined the current status and future development trends of scientific production in this field, overcoming the shortcomings of previous literature reviews that rarely comprehensively analyzed the research results from multiple perspectives and revealed the potential changes.

The annual scientific output results showed that the number of articles in the field of farmland abandonment fluctuated and increased rapidly, especially after 2016. According to the number of articles issued, the research period can be divided into three stages: budding stage, development stage, and active stage. Judging from the average number of citations of papers, the years with the strongest development in the field of farmland abandonment were 1987, 2003, 2008, and 2015. Over time, more attention was paid to abandoned farmland as such, with most papers being published from 2007 to 2017; however, the overall quality of the publications declined after 2018.

Journal source analysis showed that *Land Use Policy* was the most productive journal in the field of farmland abandonment from 1980 to 2021, with the highest number of citations and the highest H index. In terms of research strength, China was the country that published the most articles, although countries in Europe were more influential. Cooperation between countries suggested that some countries in Europe, such as Germany, the UK, and the Netherlands, focused more on collaborative research. When considering both the number of papers published by independent authors and international cooperations, papers published by countries such as China and the United States through independent research were more common.

Studies on farmland abandonment were two-branched: one branch mainly analyzed the social factors, such as policies and trade, which may promote or inhibit the abandonment of farmland; the other focused on the ecological impacts of farmland abandonment, such as impacts on soil quality, carbon sequestration, and biodiversity. The same trend was found in the authors’ collaborative network, with five of the seven most productive research groups focusing on ecological aspects and the other two on social factors.

The most common keywords were farmland abandonment, land use, vegetation, dynamics, forest, and management. Analysis of the theme evolution of abandoned farmland showed that vegetation, landscape, dynamic degree, and management were the most commonly used keywords in the first stage. The influence of the most used keywords in the second stage was weakened; in this stage, the focus was on the improvement of the extraction technology. The last period was related to the driving forces and determinants of farmland abandonment, in addition to vegetation succession and biodiversity. The theme that remained constant throughout the study period was the succession of vegetation after farmland abandonment.

This study has two limitations. First, it relies more on dynamic databases that are constantly updated as the number of indexed journals increases or decreases. Therefore, the bibliometric analysis of farmland abandonment may change in the next few years. Another limitation is that this study largely relies on the results obtained from visual charts. However, the display of results lacks a richer global perspective. The global perspective can, however, be improved by incorporating other databases, such as Scopus.

Overall, the results obtained can help researchers determine the specific research areas and contents of farmland abandonment. The analysis of the cooperation between the author and the country may facilitate the investigation of the authors in the field of farmland abandonment or the formulation of policies at the national level. In addition, this study provides a database and analyzes the different topics and methods for future researchers, assisting in the selection of research areas and topics.

According to the results, farmland abandonment research needs to be further expanded in the following aspects:

(1) In terms of data sources, farmland abandonment experienced a process from the traditional family survey data to the remote sensing image data; the latter underwent a transformation from low to high spatial resolution. However, the high-resolution images used for farmland abandonment generally have a low temporal resolution. In the future, the use of multi-source remote sensing data to combine high temporal resolution and high spatial resolution for farmland abandonment extraction may become the mainstream of research. Nowadays, there are more and more applications of hyperspectral images, and improvements in spectrum number and spectral resolution can significantly enrich spectral information, revealing more details of ground objects. Such an approach would greatly improve the accuracy of remote sensing in the identification of ground objects. In the future, this direction can be explored with the use of hyperspectral images to extract abandoned farmland.

(2) To extract abandoned farmland, change detection methods are mostly used. The extraction accuracy largely depends on the classification accuracy, and the classification error of each temporary phase will be accumulated, affecting the final result. Moreover, the classification of heterogeneous images is independent and does not consider the correlation of time, making it easy to ignore vegetation changes in the growth cycle, resulting in unreasonable change detection results. Classification time series data can effectively avoid this problem, and models such as the Landsat-based detection of trends in disturbance and recovery (LandTrendr), breaks for additive season and trend (BFAST), and continuous change detection and classification (CCDC) can demonstrate this. Today, only the LandTrendr model is used for the extraction of abandoned farmland. With the rise of deep learning, the remote sensing time series change detection research based on deep learning has attracted the interest of many researchers. Deep learning is an automated learning based on the end-to-end mechanism. It does not rely on prior knowledge, but it is still difficult to automatically mine data and acquire the spatiotemporal features of images. In the future, how to use these models to effectively extract abandoned farmland may be an important development trend.

(3) Since the 21st century, the impact of global climate change on ecology and the social economy has been an important topic. In recent years, the global outbreak of COVID-19 and regional armed conflict have caused unprecedented crises in the world, seriously affecting agriculture, the economy, human health, and food security. It is, therefore, of great significance to meet the global food crisis by rationally reclaiming abandoned farmland to rapidly increase food production and the global grain supply. However, there is still controversy about the economic cost of abandoned farmland reclamation and the ecological consequences of such reclamation. Should the abandoned farmland be reclaimed, continue to expand, or be subjected to intensive farming? Research around this area may be a future focus.

(4) According to the results of this study, future studies will still focus on the ecological impacts of abandoned fields, such as vegetation succession, carbon sequestration, and impacts on biodiversity. However, most of the current studies focus on a certain country and region, with conflicting ecological impacts along with different geographical locations and environmental conditions. At present, the ecological impact of abandoned farmland is still controversial among scholars. In the future, further discussions on this impact are: Is this a threat or an opportunity? Is this negative or positive? The formulation of policies according to local conditions when facing farmland abandonment may be an important research topic in the near future.

## Figures and Tables

**Table 1:** Journal sources with the most published articles on farmland abandonment from 1980 to 2021 (Note: TC = total citations).

Rank	Total	Articles	H Index	TC
1	*Land Use Policy*	32	17	1856
2	*Sustainability*	26	12	423
3	*Catena*	25	14	1462
4	*Land Degradation & Development*	24	14	690
5	*Land*	24	9	190
6	*Agriculture Ecosystems & Environment*	23	14	1170
7	*Forest Ecology and Management*	23	17	970
8	*Science of the Total Environment*	23	13	527
9	*Remote Sensing*	14	7	165
10	*Ecological Engineering*	13	10	357

**Table 2:** Top 10 most relevant authors in the field of farmland abandonment from 1980 to 2021 (Note: AF = articles fractionalized; TC = total citations).

Rank	Authors	Articles	AF	H Index	TC
1	Guobin Liu	19	4.12	12	789
2	Teodoro Lasanta	18	4.28	12	949
3	Tobias Kuemmerle	16	2.8	15	1813
4	Estela Nadal-Romero	15	3.43	9	359
5	Alexander V Prishchepov	15	2.6	12	1505
6	Daniel Muller	12	2.92	12	1197
7	Sha Xue	12	2.63	8	527
8	Artemi Cerda	11	3.16	10	612
9	Volker C Radeloff	11	1.78	11	1453
10	Ju-Ying Jiao	9	1.83	7	229

**Table 3:** Numbers of publications and citations on farmland abandonment in different countries from 1980 to 2021.

Country	Articles	Total Citations	Average Citations
China	193	3997	20.71
USA	105	3936	37.49
Spain	83	2856	34.41
Italy	42	1223	29.12
Germany	39	2325	59.62
Japan	34	484	14.24
Poland	30	423	14.1
Canada	26	990	38.08
Netherlands	25	2422	96.88
Australia	20	1164	58.2
United Kingdom	18	1021	56.72
France	17	723	42.53
Estonia	16	418	26.12
Sweden	15	833	55.53
Switzerland	14	1331	95.07
Russia	13	61	4.69
Greece	12	376	31.33
Slovakia	12	146	12.17
Korea	11	81	7.36
Portugal	11	310	28.18

**Table 5:** Evolution of methods for the extraction of abandoned farmland.

Countries and Regions	Data Source	Research Methods	Notes
Cal Rodo catchment (southern margin of the Pyrenees)	Two aerial photos from 1957 and 1996 (20 m)	Visual interpretation	Poyatos et al., 2003
Swiss mountains	Land use survey data during the 1980s and 1990s	Field survey	Gellrich and Zimmermann, 2006
Peyne in France	Aerial photos (1 m) from 1946, 1954, 1970, 1971, 1983, and 1988, field geographic data	Field survey, visual interpretation	Sluiter and Jong, 2006
Ijuez River Valley (Central Spanish Pyrenees)	Aerial photos from 1957, 1977, and 2002 (1 m)	Visual interpretation	Pueyo and Beguería, 2007
Poland, Slovakia, and Ukraine	Landsat TM/ETM+ images from 1986, 1988, and 2000 (30 m)	Support Vector Machines	Kuemmerle et al., 2008
Galicia (Spain)	Aerial photographs from 1956 and 1957, and land use of the plot size specified in SIGPAC (2004)	Visual interpretation	Corbelle et al., 2011
Western Ukraine	Landsat TM images from 1986, 1989, 2006, and 2008 (30 m)	Support Vector Machines	Baumann et al., 2011
Smolensk, Kaluga, Tula, Rjazan, and Vladimir in European Russia	Landsat TM/ETM+ satellite images from 1990 and 2000 (30 m)	Support Vector Machines	Prishchepov et al., 2012
Baltic countries, Belarus, and Poland	MODIS NDVI time series from 2003 to 2008 (250 m)	Vegetation index change detection, Support Vector Machines	Alcantara et al., 2012
Belarus, Lithuania, and Poland	Landsat TM/ETM+ satellite images from 1989 and 1999 (30 m)	Support Vector Machines	Prishchepov et al., 2012
Poland, Belarus, Latvia, Lithuania, and European Russia	Landsat TM/ETM+ satellite images from 1990 and 2000 (30 m)	Support Vector Machines	Prishchepov et al., 2012
Covering 6.4 Mkm^2^ across central and eastern Europe and the Balkan Peninsula, including 30 countries fully or partly	MODIS NDVI time series from 2004 to 2006 (250 m)	Vegetation index change detection, Support Vector Machines	Alcantara et al., 2013
European Russia, Ukraine, and Belarus	GLC2000, MODIS, national sown area statistics (1 km)	A spatial allocation model was developed to allocate national area statistics to remote sensing image data	Schierhorn et al., 2013
Throughout Europe	MODIS NDVI time series from 2000 to 2012 (250 m)	Vegetation index change detection, Random Forest	Estel et al., 2015
Mountainous areas in China	Household survey data of 262 counties from 2011 to 2012	Field survey	Li et al., 2017
Parts of Georgia and the North Caucasian Federal District of Russia	Landsat images from 1985 to 2015 (30 m)	LandTrendr time series change detection, object-oriented classification	Yin et al., 2018
Northern Kazakhstan	Landsat images from 1988 to 2013 (30 m)	LandTrendr time series change detection, Random Forest	Dara et al., 2018
14 regions in the world (Iraq, Nebraska, Shaanxi, Orenburg, Uganda, Belarus, Bosnia and Herzegovina, Sardinia, Volgograd, Wisconsin, Chongqing, Goias, Mato Grosso, Nepal)	Landsat images from 1987 to 2017 (30 m)	Time series change detection, Random Forest	Yin et al., 2020
Global	CCI-LC data from 1992 and 2015 (300 m)	Change detection after classification	Næss et al., 2021

## Data Availability

All relevant data sets are mentioned in the manuscript.

## Supplementary Materials

The following supporting information can be downloaded at: https://www.mdpi.com/article/10.3390/ijerph192316007/s1, Table S1: Database Article; Table S2: Most Global Cited Documents 100 (theme); Table S3: Most Global Cited Documents 100 (title).

## Appendix B

**Table A1:** Most relevant keywords when searching for publications in farmland abandonment from 1980 to 2021.

Rank	Author Keywords (DE)	Articles	Keywords-Plus (ID)	Articles
1	Abandonment/land abandonment/farmland Abandonment/abandoned farmland/cropland abandonment/abandoned land/abandoned cropland/agricultural abandonment/agricultural land abandonment/abandoned agricultural land	284	Farmland abandonment/agricultural land abandonment	107
2	Land use change	81	Vegetation	90
3	Land use	42	Dynamics	86
4	Secondary succession	32	Forest	81
5	Afforestation	25	Management	79
6	Soil erosion	24	Diversity	74
7	Remote sensing	22	Europe	62
8	Agriculture	21	Consequences	61
9	China	21	Conservation	61
10	Loess plateau	21	Biodiversity	56
11	Succession	20	Landscape	54
12	Mediterranean	15	Patterns	54
13	Soil organic carbon	15	Drivers	46
14	Biodiversity	14	Restoration	46
15	Reforestation	14	Impact	44
16	Restoration	14	Cover	43
17	Spain	14	Nitrogen	43
18	Europe	11	Carbon	42
19	GIS	11	Succession	40
20	Natural regeneration	11	Soil erosion	39
