# Supplementary material for: Status, Trend, and Prospect of Global Farmland Abandonment Research: A Bibliometric Analysis
Source: Int J Environ Res Public Health. 2022 Nov 30;19(23):16007. doi: 10.3390/ijerph192316007 (PMC9735913; doi:10.3390/ijerph192316007)
Supplement: Supplementary file 1 [file ijerph-19-16007-s001.zip › ijerph-2052520-supplementary.pdf]

Supplementary Table S1 Database Article

Sequence	Paper	DOI
1	CRAMER VA, 2008, TRENDS ECOL EVOL	10.1016/j.tree.2007.10.005
2	VERBURG PH, 2009, LANDSCAPE ECOL	10.1007/s10980-009-9355-7
3	GEHRIG-FASEL J, 2007, J VEG SCI	10.1658/1100-9233(2007)18[571:TLSITS]2.0.CO;2
4	CAMPBELL JE, 2008, ENVIRON SCI TECHNOL	10.1021/es800052w
5	SILVER WL, 2000, RESTOR ECOL	10.1046/j.1526-100x.2000.80054.x
6	GELLRICH M, 2007, AGR ECOSYST ENVIRON	10.1016/j.agee.2006.05.001
7	GARCIA-RUIZ JM, 2011, AGR ECOSYST ENVIRON	10.1016/j.agee.2011.01.003
8	BAUMANN M, 2011, LAND USE POLICY	10.1016/j.landusepol.2010.11.003
9	QUEIROZ C, 2014, FRONT ECOL ENVIRON	10.1890/120348
10	RENWICK A, 2013, LAND USE POLICY	10.1016/j.landusepol.2012.04.005
11	ESTEL S, 2015, REMOTE SENS ENVIRON	10.1016/j.rse.2015.03.028
12	PRISHCHEPOV AV, 2013, LAND USE POLICY	10.1016/j.landusepol.2012.06.011
13	POYATOS R, 2003, MT RES DEV	10.1659/0276-4741(2003)023[0362:LUALCC]2.0.CO;2
14	ZHANG C, 2016, SOIL BIOL BIOCHEM	10.1016/j.soilbio.2016.02.013
15	KOULOURI M, 2007, CATENA	10.1016/j.catena.2006.07.001
16	LASANTA T, 2017, CATENA	10.1016/j.catena.2016.02.024
17	LUGO AE, 2004, FOREST ECOL MANAG	10.1016/j.foreco.2003.09.012
18	KUEMMERLE T, 2008, ECOSYSTEMS	10.1007/s10021-008-9146-z
19	BOWEN ME, 2007, BIOL CONSERV	10.1016/j.biocon.2007.08.012
20	ROMERO-CALCERRADA R, 2004, LANDSCAPE URBAN PLAN	10.1016/S0169-2046(03)00112-9
21	GELLRICH M, 2007, LANDSCAPE URBAN PLAN	10.1016/j.landurbplan.2006.03.004
22	VAN DER WAL A, 2006, SOIL BIOL BIOCHEM	10.1016/j.soilbio.2005.04.017
23	SUAREZ-SEOANE S, 2002, BIOL CONSERV	10.1016/S0006-3207(01)00213-0
24	MULLER D, 2013, AGR SYST	10.1016/j.agsy.2012.12.010
25	HOOPER E, 2005, J APPL ECOL	10.1111/j.1365-2664.2005.01106.x
26	ALCANTARA C, 2013, ENVIRON RES LETT	10.1088/1748-9326/8/3/035035
27	LASANTA T, 2000, CATENA	10.1016/S0341-8162(99)00079-X
28	DUNJO G, 2003, CATENA	10.1016/S0341-8162(02)00148-0
29	CAMMERAAT LH, 1999, CATENA	10.1016/S0341-8162(98)00072-1
30	BEILIN R, 2014, LAND USE POLICY	10.1016/j.landusepol.2013.07.003
31	PRISHCHEPOV AV, 2012, ENVIRON RES LETT	10.1088/1748-9326/7/2/024021
32	CHAUCHARD S, 2007, ECOSYSTEMS	10.1007/s10021-007-9065-4
33	GOUGH MW, 1990, BIOL CONSERV	10.1016/0006-3207(90)90104-W
34	HUNZIKER M, 1995, LANDSCAPE URBAN PLAN	10.1016/0169-2046(95)93251-J
35	HEDLUND K, 2003, OIKOS	10.1034/j.1600-0706.2003.12511.x
36	TERRES JM, 2015, LAND USE POLICY	10.1016/j.landusepol.2015.06.009
37	ZHANG Y, 2014, LAND USE POLICY	10.1016/j.landusepol.2014.05.011
38	SLUITER R, 2007, LANDSCAPE ECOL	10.1007/s10980-006-9049-3

Sequence	Paper	DOI
39	HOOPER E, 2002, ECOL APPL	10.1890/1051-0761(2002)012[1626:RONTST]2.0.CO;2
40	LI SF, 2017, J GEOGR SCI	10.1007/s11442-017-1426-0
41	LESSCHEN JP, 2008, EARTH SURF PROC LAND	10.1002/esp.1676
42	WANG B, 2011, ENVIRON EARTH SCI	10.1007/s12665-010-0577-4
43	KUEMMERLE T, 2011, GLOBAL CHANGE BIOL	10.1111/j.1365-2486.2010.02333.x
44	MOLINILLO M, 1997, ENVIRON MANAGE	10.1007/s002679900051
45	LABRECQUE M, 2003, BIOMASS BIOENERG	10.1016/S0961-9534(02)00192-7
46	LIU Y, 2012, GEOMORPHOLOGY	10.1016/j.geomorph.2011.10.009
47	PLIENINGER T, 2014, PLOS ONE	10.1371/journal.pone.0098355
48	ARNAEZ J, 2011, LAND DEGRAD DEV	10.1002/ldr.1032
49	SCHIERHORN F, 2013, GLOBAL BIOGEOCHEM CY	10.1002/2013GB004654
50	XU DD, 2019, J ENVIRON MANAGE	10.1016/j.jenvman.2018.11.136
51	SIRAMI C, 2008, BIOL CONSERV	10.1016/j.biocon.2007.10.015
52	CERDA A, 1997, ARID SOIL RES REHAB	10.1080/15324989709381469
53	DIAZ GI, 2011, LANDSCAPE URBAN PLAN	10.1016/j.landurbplan.2010.11.005
54	LASANTA T, 2015, ENVIRON SCI POLICY	10.1016/j.envsci.2015.05.012
55	MUNROE DK, 2013, CURR OPIN ENV SUST	10.1016/j.cosust.2013.06.010
56	UCHIDA K, 2014, ECOL MONOGR	10.1890/13-2170.1
57	HOOGWIJK M, 2009, BIOMASS BIOENERG	10.1016/j.biombioe.2008.04.005
58	LESSCHEN JP, 2007, CATENA	10.1016/j.catena.2006.05.014
59	CHAPMAN CA, 1999, CONSERV BIOL	10.1046/j.1523-1739.1999.98229.x
60	YAN JZ, 2016, LAND USE POLICY	10.1016/j.landusepol.2016.06.014
61	HATNA E, 2011, ECOSYSTEMS	10.1007/s10021-011-9441-y
62	HOLTKAMP R, 2008, APPL SOIL ECOL	10.1016/j.apsoil.2007.11.002
63	ZELLER V, 2001, SOIL BIOL BIOCHEM	10.1016/S0038-0717(00)00208-X
64	SIRAMI C, 2007, DIVERS DISTRIB	10.1111/j.1472-4642.2006.00297.x
65	PRISHCHEPOV AV, 2012, REMOTE SENS ENVIRON	10.1016/j.rse.2012.08.017
66	DUARTE F, 2008, J ENVIRON MANAGE	10.1016/j.jenvman.2007.05.024
67	MEYFROIDT P, 2016, GLOBAL ENVIRON CHANG	10.1016/j.gloenvcha.2016.01.003
68	LEVERS C, 2018, SCI TOTAL ENVIRON	10.1016/j.scitotenv.2018.06.326
69	CHERRY DS, 2001, ENVIRON POLLUT	10.1016/S0269-7491(00)00093-2
70	GIBSON CWD, 1987, BIOL CONSERV	10.1016/0006-3207(87)90132-7
71	YIN H, 2018, REMOTE SENS ENVIRON	10.1016/j.rse.2018.02.050
72	BENJAMIN K, 2005, LANDSCAPE ECOL	10.1007/s10980-005-0068-2
73	PREVOSTO B, 2011, FOLIA GEOBOT	10.1007/s12224-010-9096-z
74	ZHANG KR, 2010, FOREST ECOL MANAG	10.1016/j.foreco.2010.02.014
75	BENAYAS JMR, 2005, FOREST ECOL MANAG	10.1016/j.foreco.2005.03.032
76	ROMERO-DIAZ A, 2017, CATENA	10.1016/j.catena.2016.08.013
77	ZHANG JT, 2005, J ARID ENVIRON	10.1016/j.jaridenv.2005.03.027
78	PICHTEL JR, 1994, J ENVIRON QUAL	10.2134/jeq1994.00472425002300040022x
79	SANCHEZ-CUERVO AM, 2013, ECOSYSTEMS	10.1007/s10021-013-9667-y

Sequence	Paper	DOI
80	MEINERS SJ, 2001, ECOGRAPHY	10.1034/j.1600-0587.2001.240602.x
81	SPERA SA, 2014, ENVIRON RES LETT	10.1088/1748-9326/9/6/064010
82	PAZUR R, 2014, APPL GEOGR	10.1016/j.apgeog.2014.07.014
83	JOHANSSON T, 1999, BIOMASS BIOENERG-a	10.1016/S0961-9534(98)00075-0
84	WANG B, 2013, EARTH SURF PROC LAND	10.1002/esp.3459
85	LESSCHEN JP, 2008, J ARID ENVIRON	10.1016/j.jaridenv.2008.06.006
86	GARCIA-RUIZ JM, 2005, CATENA	10.1016/j.catena.2004.05.006
87	CERDA A, 2018, PROG PHYS GEOG	10.1177/0309133318758521
88	COLON SM, 2006, BIOTROPICA	10.1111/j.1744-7429.2006.00159.x
89	HOOPER ER, 2004, ECOLOGY	10.1890/03-0655
90	KAUFFMAN JB, 2009, ECOL APPL	10.1890/08-1696.1
91	HARMER R, 2001, BIOL CONSERV	10.1016/S0006-3207(01)00072-6
92	XIE HL, 2014, SUSTAINABILITY-BASEL	10.3390/su6031260
93	NOVARA A, 2016, SCI TOTAL ENVIRON	10.1016/j.scitotenv.2016.01.095
94	NOVARA A, 2017, SCI TOTAL ENVIRON	10.1016/j.scitotenv.2016.10.123
95	NADAL-ROMERO E, 2016, AGR ECOSYST ENVIRON	10.1016/j.agee.2016.05.003
96	CORBELLE-RICO E, 2012, LAND USE POLICY	10.1016/j.landusepol.2011.08.008
97	HARDEN CP, 1996, MT RES DEV	10.2307/3673950
98	GISPERT M, 2013, GEODERMA	10.1016/j.geoderma.2013.03.012
99	PRICE B, 2015, APPL GEOGR	10.1016/j.apgeog.2014.12.009
100	SIKOR T, 2009, WORLD DEV	10.1016/j.worlddev.2008.08.013
101	VASSILEV K, 2011, PLANT BIOSYST	10.1080/11263504.2011.601337
102	SHANG ZH, 2008, LAND DEGRAD DEV	10.1002/ldr.861
103	MULLER D, 2008, ANN ASSOC AM GEOGR	10.1080/00045600802262323
104	URI V, 2002, FOREST ECOL MANAG	10.1016/S0378-1127(01)00478-9
105	GRADINARU SR, 2015, ECOL INDIC	10.1016/j.ecolind.2015.05.009
106	KNOKE T, 2014, NAT COMMUN	10.1038/ncomms6612
107	UEMATSU Y, 2010, AGR ECOSYST ENVIRON	10.1016/j.agee.2009.10.010
108	PUEYO Y, 2007, LANDSCAPE URBAN PLAN	10.1016/j.landurbplan.2007.04.008
109	CREMASCHI M, 2006, QUATERN INT	10.1016/j.quaint.2006.01.020
110	SHEFFER E, 2012, ANN FOREST SCI	10.1007/s13595-011-0181-0
111	MALJANEN M, 2007, BOREAL ENVIRON RES	NA
112	HACKWORTH J, 2014, PROG PLANN	10.1016/j.progress.2013.03.004
113	DENG L, 2016, ECOL ENG	10.1016/j.ecoleng.2016.01.086
114	WEISSTEINER CJ, 2011, GLOBAL PLANET CHANGE	10.1016/j.gloplacha.2011.07.009
115	DARA A, 2018, REMOTE SENS ENVIRON	10.1016/j.rse.2018.05.005
116	USTAOGLU E, 2018, ENVIRON REV	10.1139/er-2018-0001
117	MOUILLOT F, 2005, LANDSCAPE ECOL	10.1007/s10980-004-1297-5
118	DENG L, 2013, PLOS ONE	10.1371/journal.pone.0071923
119	FOOTE RL, 2010, ECOSYSTEMS	10.1007/s10021-010-9355-0
120	OTERO I, 2015, ECOL SOC	10.5751/ES-07378-200207
121	RODRIGO-COMINO J, 2018, PEDOSPHERE	10.1016/S1002-0160(17)60441-7

Sequence	Paper	DOI
122	KOLECKA N, 2017, APPL GEOGR	10.1016/j.apgeog.2017.09.002
123	MENKIS A, 2006, PLANT PATHOL	10.1111/j.1365-3059.2005.01295.x
124	SMALIYCHUK A, 2016, GLOBAL ENVIRON CHANG	10.1016/j.gloenvcha.2016.02.009
125	SALIFU KF, 2009, RESTOR ECOL	10.1111/j.1526-100X.2008.00373.x
126	DEININGER K, 2012, WORLD DEV	10.1016/j.worlddev.2012.05.010
127	RUSKULE A, 2013, LANDSCAPE URBAN PLAN	10.1016/j.landurbplan.2013.03.012
128	SKOUSEN JG, 1994, J ENVIRON QUAL	10.2134/jeq1994.00472425002300060015x
129	TRUAX B, 2012, FOREST ECOL MANAG	10.1016/j.foreco.2011.12.012
130	HAASE P, 1997, J VEG SCI	10.2307/3237366
131	KORTHALS GW, 2001, FUNCT ECOL	10.1046/j.0269-8463.2001.00551.x
132	LANA-RENAULT N, 2009, EARTH SURF PROC LAND	10.1002/esp.1825
133	GONZALEZ JE, 1994, FOREST ECOL MANAG	NA
134	PALOMBO C, 2013, PLANT BIOSYST	10.1080/11263504.2013.772081
135	SANDERSON FJ, 2013, AGR ECOSYST ENVIRON	10.1016/j.agee.2013.01.015
136	JIANG JP, 2009, PEDOSPHERE	10.1016/S1002-0160(09)60169-7
137	LEMENIH M, 2004, FOREST ECOL MANAG	10.1016/j.foreco.2004.02.055
138	CAMMERAAT ELH, 2010, ECOHYDROLOGY	10.1002/eco.161
139	KOU M, 2016, LAND DEGRAD DEV	10.1002/ldr.2356
140	SCHRODER P, 2008, ENVIRON SCI POLLUT R	10.1065/espr2008.03.481
141	BENJAMIN K, 2007, LANDSCAPE URBAN PLAN	10.1016/j.landurbplan.2007.04.009
142	RIVERA LW, 2000, PLANT ECOL	10.1023/A:1009825211430
143	URI V, 2007, EUR J FOREST RES	10.1007/s10342-007-0171-9
144	DORMAAR JF, 1985, J RANGE MANAGE	10.2307/3899737
145	LI SF, 2018, LAND DEGRAD DEV	10.1002/ldr.2924
146	WANG GL, 2009, PLANT SOIL	10.1007/s11104-008-9773-3
147	JIAO F, 2013, ECOL ENG	10.1016/j.ecoleng.2013.06.036
148	BRANDOLINI P, 2018, LAND DEGRAD DEV	10.1002/ldr.2672
149	ZHANG Y, 2016, J RURAL STUD	10.1016/j.jrurstud.2016.06.019
150	PYWELL RF, 1995, J APPL ECOL	10.2307/2405106
151	LOW F, 2015, APPL GEOGR	10.1016/j.apgeog.2015.05.009
152	KUITERS AT, 2003, FOREST ECOL MANAG	10.1016/S0378-1127(03)00136-1
153	STOVER ME, 1998, J TORREY BOT SOC	10.2307/2997302
154	ZUMKEHR A, 2013, ENVIRON SCI TECHNOL	10.1021/es3033132
155	DENG X, 2019, LAND USE POLICY	10.1016/j.landusepol.2019.104243
156	YU Z, 2018, GLOBAL ECOL BIOGEOGR	10.1111/geb.12697
157	ZHAO D, 2017, SOIL TILL RES	10.1016/j.still.2016.08.007
158	NUNES AN, 2010, LAND DEGRAD DEV	10.1002/ldr.973
159	ESCRIBANO-AVILA G, 2014, J APPL ECOL	10.1111/1365-2664.12340
160	EMRAN M, 2012, EUR J SOIL SCI	10.1111/j.1365-2389.2012.01493.x
161	KOLECKA N, 2015, REMOTE SENS-BASEL	10.3390/rs70708300
162	LOPEZ BN, 2011, ARCH ENVIRON CON TOX	10.1007/s00244-010-9590-6
163	WEI XR, 2013, BIOL FERT SOILS	10.1007/s00374-012-0754-6

Sequence	Paper	DOI
164	ALVES DS, 2003, INT J REMOTE SENS	10.1080/0143116021000015807
165	LABAUNE C, 2002, GLOBAL ECOL BIOGEOGR	10.1046/j.1466-822X.2002.00280.x
166	YELOFF D, 2007, J BIOGEOGR	10.1111/j.1365-2699.2006.01674.x
167	RAIESI F, 2012, PLANT SOIL	10.1007/s11104-011-0941-5
168	ZHANG Q, 2018, LAND USE POLICY	10.1016/j.landusepol.2018.01.001
169	JOHANSSON T, 2000, BIOMASS BIOENERG	10.1016/S0961-9534(99)00078-1
170	HAN XY, 2018, AGR ECOSYST ENVIRON	10.1016/j.agee.2018.02.006
171	JIAO JY, 2007, RESTOR ECOL	10.1111/j.1526-100X.2007.00235.x
172	LATOCHA A, 2016, CATENA	10.1016/j.catena.2016.05.027
173	URI V, 2011, ECOL ENG	10.1016/j.ecoleng.2011.01.016
174	FIGUEIREDO J, 2011, LANDSCAPE ECOL	10.1007/s10980-011-9605-3
175	ZHANG C, 2012, EUR J SOIL BIOL	10.1016/j.ejsobi.2012.01.002
176	LENDA M, 2012, P ROY SOC B-BIOL SCI	10.1098/rspb.2011.2153
177	RIES JB, 2008, CATENA	10.1016/j.catena.2007.06.001
178	GONZALEZ G, 1996, PEDOBIOLOGIA	NA
179	RYU BG, 2009, SEP SCI TECHNOL	10.1080/01496390902983778
180	KIVINEN S, 2017, SUSTAINABILITY-BASEL	10.3390/su9101705
181	SLOAN S, 2016, LANDSCAPE ECOL	10.1007/s10980-015-0267-4
182	LOW F, 2018, REMOTE SENS-BASEL	10.3390/rs10020159
183	TANG KL, 1998, CHINESE SCI BULL	10.1007/BF02883721
184	JIAO JY, 2008, PEDOSPHERE	10.1016/S1002-0160(07)60099-X
185	VAN LEEUWEN CCE, 2019, LAND USE POLICY	10.1016/j.landusepol.2019.01.018
186	BRAMBILLA M, 2007, BIRD STUDY	10.1080/00063650709461471
187	RUSKULE A, 2012, AGROFOREST SYST	10.1007/s10457-012-9495-7
188	STANDISH RJ, 2008, J APPL ECOL	10.1111/j.1365-2664.2008.01558.x
189	XU DD, 2019, LAND USE POLICY	10.1016/j.landusepol.2019.104164
190	WANG H, 2018, GEODERMA	10.1016/j.geoderma.2018.03.037
191	GABARRON-GALEOTE MA, 2015, GEODERMA	10.1016/j.geoderma.2015.03.007
192	HERRANDO S, 2016, ENVIRON CONSERV	10.1017/S0376892915000260
193	URI V, 2007, BIOMASS BIOENERG	10.1016/j.biombioe.2006.08.003
194	FRESCHET GT, 2014, ECOLOGY	10.1890/13-0824.1
195	OTERO I, 2011, LAND USE POLICY	10.1016/j.landusepol.2010.06.002
196	JOHANSSON T, 1999, SILVA FENN	10.14214/sf.649
197	LESIV M, 2018, SCI DATA	10.1038/sdata.2018.56
198	MENKIS A, 2007, MYCORRHIZA	10.1007/s00572-007-0110-0
199	TRUAX B, 2014, FORESTS	10.3390/f5123107
200	ZHANG C, 2015, ECOL ENG	10.1016/j.ecoleng.2014.11.059
201	ORLANDI S, 2016, BIODIVERS CONSERV	10.1007/s10531-016-1046-5
202	LI YY, 2015, ECOL RES	10.1007/s11284-014-1230-6
203	ZHANG W, 2019, SOIL BIOL BIOCHEM	10.1016/j.soilblo.2019.03.017
204	ALONSO-SARRIA F, 2016, LAND DEGRAD DEV	10.1002/ldr.2447
205	WITMER FDW, 2008, INT J REMOTE SENS	10.1080/01431160801891879

Sequence	Paper	DOI
206	LI JJ, 2013, J SOIL SEDIMENT	10.1007/s11368-013-0652-z
207	SCOZZAFAVA S, 2006, LANDSCAPE URBAN PLAN	10.1016/j.landurbplan.2004.10.006
208	BLAIR D, 2018, LAND-BASEL	10.3390/land7040121
209	NIKOLOV SC, 2010, BIRD CONSERV INT	10.1017/S0959270909990244
210	AGNOLETTI M, 2019, SUSTAINABILITY-BASEL	10.3390/su11010235
211	BANERJEE MJ, 2006, RESTOR ECOL	10.1111/j.1526-100X.2006.00142.x
212	DE BAETS S, 2013, SOIL USE MANAGE	10.1111/sum.12017
213	WERTEBACH TM, 2017, GLOBAL CHANGE BIOL	10.1111/gcb.13650
214	FERNANDEZ-CALVINO D, 2008, LAND DEGRAD DEV	10.1002/ldr.831
215	LOPEZ-VICENTE M, 2011, J SOIL SEDIMENT	10.1007/s11368-011-0428-2
216	REY-BENAYAS JM, 2010, FOREST ECOL MANAG	10.1016/j.foreco.2010.04.004
217	LLORENS P, 1997, EARTH SURF PROC LAND	3.0.CO;2-1"<br>target="_blank">10.1002/(SICI)1096-<br>9837(199711)22:113.0.CO;2-1
218	SHI TC, 2018, LAND USE POLICY	10.1016/j.landusepol.2017.10.039
219	SPOHN M, 2016, PLANT SOIL	10.1007/s11104-015-2513-6
220	KUHMAN TR, 2011, CAN J FOREST RES	10.1139/X11-026
221	WALTHER P, 1986, MT RES DEV	10.2307/3673371
222	ALIX-GARCIA J, 2012, LAND ECON	10.3368/le.88.3.425
223	ZARAGOZI B, 2012, AGR ECOSYST ENVIRON	10.1016/j.agee.2012.03.019
224	IOFFE G, 2012, EURASIAN GEOGR ECON	10.2747/1539-7216.53.4.527
225	BENAYAS JMR, 2004, FOREST ECOL MANAG	10.1016/j.foreco.2004.02.035
226	JOHNSON CD, 1995, J ENVIRON QUAL	10.2134/jeq1995.00472425002400040014x
227	FILHO WL, 2017, INT J SUST DEV WORLD	10.1080/13504509.2016.1240113
228	GIRAUDOUX P, 1994, ACTA OECOL	NA
229	SANZ ASR, 2013, ECOL SOC	10.5751/ES-05556-180238
230	URI V, 2003, FOREST ECOL MANAG	10.1016/S0378-1127(03)00210-X
231	ALBERTI G, 2011, REG ENVIRON CHANGE	10.1007/s10113-011-0229-6
232	GELLRICH M, 2007, ENVIRON MODEL ASSESS	10.1007/s10666-006-9062-6
233	DONG JW, 2011, ENVIRON MONIT ASSESS	10.1007/s10661-010-1724-9
234	HERRANDO S, 2014, ECOL INDIC	10.1016/j.ecolind.2014.04.011
235	JOHANSSON T, 2007, FORESTRY	10.1093/forestry/cpl049
236	DENG X, 2018, INT J ENV RES PUB HE	10.3390/ijerph15091808
237	COGLIASTRO A, 1997, FOREST ECOL MANAG	10.1016/S0378-1127(97)00042-X
238	KATAYAMA N, 2015, AGR ECOSYST ENVIRON	10.1016/j.agee.2015.08.014
239	WANG J, 2019, SOIL TILL RES	10.1016/j.still.2019.104305
240	WOODS K, 2004, J TROP FOR SCI	NA
241	ZAKKAK S, 2015, J ENVIRON MANAGE	10.1016/j.jenvman.2015.09.005
242	UCHIDA K, 2015, J APPL ECOL	10.1111/1365-2664.12443
243	WITMER FDW, 2009, ANN ASSOC AM GEOGR	10.1080/00045600903260697
244	URI V, 2009, FORESTRY	10.1093/forestry/cpn040
245	ORLOWSKI G, 2005, AGR ECOSYST ENVIRON	10.1016/j.agee.2005.06.012

Sequence	Paper	DOI
246	WEI XR, 2012, PLOS ONE	10.1371/journal.pone.0032054
247	BOCIO I, 2004, ANN FOREST SCI	10.1051/forest:2004009
248	ZAKKAK S, 2014, J NAT CONSERV	10.1016/j.jnc.2013.11.001
249	SU GD, 2018, SUSTAINABILITY-BASEL	10.3390/su10103676
250	HAN Z, 2019, J CLEAN PROD	10.1016/j.jclepro.2019.117888
251	BRINKERT A, 2016, BIODIVERS CONSERV	10.1007/s10531-015-1020-7
252	GRANTZ DA, 1998, J ENVIRON QUAL	10.2134/jeq1998.00472425002700040033x
253	GABARRON-GALEORE MA, 2015, AGR ECOSYST ENVIRON	10.1016/j.agee.2014.08.027
254	LARSSON S, 2005, BIOMASS BIOENERG	10.1016/j.biombioe.2004.05.003
255	YUAN ZQ, 2016, SCI TOTAL ENVIRON	10.1016/j.scitotenv.2015.09.108
256	LIANG Y, 2020, J HYDROL	10.1016/j.jhydrol.2020.124694
257	PEDRINI P, 2001, BIRD STUDY	10.1080/00063650109461218
258	GRADINARU SR, 2019, ECOL INDIC	10.1016/j.ecolind.2017.06.022
259	HERBERT DA, 2003, BIOGEOCHEMISTRY	10.1023/A:1026020210887
260	SOJNEKOVA M, 2015, ECOL ENG	10.1016/j.ecoleng.2015.01.042
261	YIN H, 2020, REMOTE SENS ENVIRON	10.1016/j.rse.2020.111873
262	DODDS WK, 1996, SOIL BIOL BIOCHEM	10.1016/0038-0717(96)00057-0
263	KURGANOVA IN, 2007, EURASIAN SOIL SCI+	10.1134/S1064229307010085
264	LI JW, 2019, SCI TOTAL ENVIRON	10.1016/j.scitotenv.2019.133613
265	ROBLEDANO-AYMERICH F, 2014, AGR ECOSYST ENVIRON	10.1016/j.agee.2014.08.006
266	ZHANG W, 2018, ECOL ENG	10.1016/j.ecoleng.2018.07.031
267	AZEVEDO JC, 2011, LANDSCAPE ECOLOGY IN FOREST MANAGEMENT AND CONSERVATION: CHALLENGES AND SOLUTIONS FOR GLOBAL CHANGE	NA
268	BENAYAS JMR, 2003, APPL VEG SCI	10.1111/j.1654-109X.2003.tb00582.x
269	NADAL-ROMERO E, 2016, SCI TOTAL ENVIRON	10.1016/j.scitotenv.2016.05.031
270	ZHANG C, 2017, ECOL APPL	10.1002/eap.1598
271	SIL A, 2019, ECOSYST SERV	10.1016/j.ecoser.2019.100908
272	SHI TC, 2016, SUSTAINABILITY-BASEL	10.3390/su8100988
273	KOSMAS C, 2015, CATENA	10.1016/j.catena.2014.02.006
274	HE SX, 2016, CATENA	10.1016/j.catena.2015.01.027
275	URI V, 2008, FOREST ECOL MANAG	10.1016/j.foreco.2007.09.019
276	SHOPE CL, 2006, APPL GEOCHEM	10.1016/j.apgeochem.2005.11.004
277	ESCRIBANO-AVILA G, 2012, PLOS ONE	10.1371/journal.pone.0046993
278	CHANG XF, 2017, LAND DEGRAD DEV	10.1002/ldr.2679
279	ALLEN EB, 2005, ISR J PLANT SCI	10.1560/65LM-55YH-GB49-5BJM
280	JOHANSSON T, 1999, BIOMASS BIOENERG	10.1016/S0961-9534(99)00073-2
281	MARUSHIA RG, 2011, RESTOR ECOL	10.1111/j.1526-100X.2009.00540.x
282	JANUS J, 2019, ECOL ENG	10.1016/j.ecoleng.2019.06.017
283	LOCKWELL J, 2012, PLANT SOIL	10.1007/s11104-012-1251-2
284	LLOVET J, 2009, INT J WILDLAND FIRE	10.1071/WF07089
285	BONANOMI G, 2013, BIODIVERS CONSERV	10.1007/s10531-013-0502-8

Sequence	Paper	DOI
286	LANA-RENAULT N, 2018, CUAD INVESTIG GEOGR	10.18172/cig.3475
287	SOCHACKI SJ, 2012, GCB BIOENERGY	10.1111/j.1757-1707.2011.01139.x
288	CHENG ZB, 2018, SCI TOTAL ENVIRON	10.1016/j.scitotenv.2018.02.259
289	TOMAZ C, 2013, FOREST ECOL MANAG	10.1016/j.foreco.2013.07.044
290	WANG C, 2016, SCI REP-UK	10.1038/srep37658
291	LUCAS-BORJA ME, 2019, WATER-SUI	10.3390/w11030503
292	CUESTA B, 2012, ACTA OECOL	10.1016/j.actao.2011.09.004
293	VAN DER ZANDEN EH, 2018, REG ENVIRON CHANGE	10.1007/s10113-018-1294-x
294	MALY S, 2000, BIOL FERT SOILS	10.1007/s003740050634
295	ABOLINA E, 2015, LAND USE POLICY	10.1016/j.landusepol.2015.08.022
296	HOU J, 2014, GLOBAL PLANET CHANGE	10.1016/j.gloplacha.2013.12.008
297	LOPEZ-SANGIL L, 2011, BIOL FERT SOILS	10.1007/s00374-010-0510-8
298	HAGEMAN PL, 2000, ICARD 2000, VOLS I AND II, PROCEEDINGS	NA
299	FETZEL T, 2014, ECOL ECON	10.1016/j.ecolecon.2013.12.002
300	RUSSELL WB, 1986, CAN J BOT	10.1139/b86-177
301	SHAO JA, 2016, J GEOGR SCI	10.1007/s11442-016-1263-6
302	GUILHERME JL, 2013, PLOS ONE	10.1371/journal.pone.0073619
303	OHTSUKA T, 1999, ECOL RES	10.1046/j.1440-1703.1999.143304.x
304	NEWMASTER SG, 2006, CAN J FOREST RES	10.1139/X06-021
305	CAVANI L, 2016, J ENVIRON MANAGE	10.1016/j.jenvman.2016.07.050
306	MILENOV P, 2014, INT J APPL EARTH OBS	10.1016/j.jag.2014.03.013
307	CASTRO H, 2010, PLANT SOIL	10.1007/s11104-010-0333-2
308	GAMMONS CH, 2007, ENVIRON GEOL	10.1007/s00254-007-0676-z
309	MUNTEANU C, 2017, REG ENVIRON CHANGE	10.1007/s10113-016-1097-x
310	DEL PLIEGO PG, 2016, BIOL CONSERV	10.1016/j.biocon.2016.07.038
311	MALAVASI M, 2018, REG ENVIRON CHANGE	10.1007/s10113-018-1368-9
312	CASTILLO CP, 2020, SUSTAINABILITY-BASEL	10.3390/su12020560
313	PENA-ANGULO D, 2019, CATENA	10.1016/j.catena.2019.05.010
314	SIIPILEHTO J, 2001, SILVA FENN	10.14214/sf.577
315	PAZUR R, 2020, LAND-BASEL	10.3390/land9090316
316	LIU AY, 2013, HEALTH PLACE	10.1016/j.healthplace.2013.03.012
317	DOREN RF, 1991, ENVIRON MANAGE	10.1007/BF02393843
318	HUA XB, 2016, CROP PROT	10.1016/j.cropro.2016.03.005
319	XU F, 2019, HABITAT INT	10.1016/j.habitatint.2018.12.006
320	BELL S, 2020, ENVIRON SCI POLICY	10.1016/j.envsci.2020.03.018
321	HARRIS TM, 2016, J CLEAN PROD	10.1016/j.jclepro.2015.09.057
322	KNOKE T, 2013, GLOBAL ENVIRON CHANG	10.1016/j.gloenvcha.2013.07.004
323	JIA GM, 2010, PEDOSPHERE	10.1016/S1002-0160(10)60024-0
324	FONTI P, 2006, J VEG SCI	10.1658/1100-9233(2006)017[0103:TRSCDI]2.0.CO;2
325	CELIS G, 2011, FOREST ECOL MANAG	10.1016/j.foreco.2010.10.005
326	PEPE G, 2019, WATER-SUI	10.3390/w11081552

Sequence	Paper	DOI
327	SCHALDACH R, 2007, REG ENVIRON CHANGE	10.1007/s10113-007-0034-4
328	TREML V, 2016, J VEG SCI	10.1111/jvs.12448
329	WILSON CA, 2006, J ENVIRON MONITOR	10.1039/b516614d
330	KAMP J, 2018, AGR ECOSYST ENVIRON	10.1016/j.agee.2018.09.009
331	CORBELLE-RICO E, 2014, LAND USE POLICY	10.1016/j.landusepol.2013.10.013
332	CHENG ZB, 2019, SOIL TILL RES	10.1016/j.still.2018.12.015
333	TRIGALET S, 2016, GEODERMA	10.1016/j.geoderma.2016.01.014
334	WANG HL, 2017, SCI TOTAL ENVIRON	10.1016/j.scitotenv.2017.07.014
335	TAKAHASHI K, 2006, FOREST ECOL MANAG	10.1016/j.foreco.2006.03.015
336	LASANTA T, 1995, PHYS CHEM EARTH	10.1016/0079-1946(95)00042-9
337	HOLMES MA, 2018, J ECOL	10.1111/1365-2745.12970
338	ALVAREZ-AYUSO E, 2016, SCI TOTAL ENVIRON	10.1016/j.scitotenv.2015.10.054
339	CERDA A, 2019, WATER-SUI	10.3390/w11040824
340	ROUNDY BA, 2001, ARID LAND RES MANAG	10.1080/153249801300000798
341	VINOGRADOVS I, 2018, AGR ECOSYST ENVIRON	10.1016/j.agee.2017.10.016
342	KERCKHOF A, 2016, SPRINGERPLUS	10.1186/s40064-016-2079-7
343	TOTH T, 1995, SOIL SCI	10.1097/00010694-199509000-00007
344	YUSOFF NM, 2017, INT J DIGIT EARTH	10.1080/17538947.2016.1216615
345	HUSAIN R, 2019, PLOS ONE	10.1371/journal.pone.0221570
346	NADAL-ROMERO E, 2018, LAND DEGRAD DEV	10.1002/ldr.2542
347	JEDLICKA P, 2007, BIOLOGIA	10.2478/s11756-007-0017-4
348	URSINO N, 2014, GEOPHYS RES LETT	10.1002/2014GL061560
349	ERIKSSON E, 2006, SILVA FENN	10.14214/sf.317
350	CATORCI A, 2013, POL J ECOL	NA
351	TIAN JH, 2017, PLANT SOIL	10.1007/s11104-017-3253-6
352	CANESSA S, 2013, ORYX	10.1017/S0030605311001542
353	BUTA M, 2019, SUSTAINABILITY-BASEL	10.3390/su11123393
354	WANG N, 2013, ECOL ENG	10.1016/j.ecoleng.2012.12.055
355	JANUS J, 2018, APPL GEOGR	10.1016/j.apgeog.2018.06.002
356	VUICHARD N, 2009, ENVIRON SCI TECHNOL	10.1021/es901652t
357	RUSKULE A, 2016, NEW FOREST	10.1007/s11056-016-9532-x
358	VACQUIE LA, 2015, J MT SCI-ENGL	10.1007/s11629-014-3405-6
359	VISOCKIENE JS, 2019, LAND USE POLICY	10.1016/j.landusepol.2019.01.013
360	KRAUSE A, 2016, EARTH SYST DYNAM	10.5194/esd-7-745-2016
361	CHAUDHARY S, 2018, SUSTAINABILITY-BASEL	10.3390/su10072331
362	CARLESI S, 2013, AGR ECOSYST ENVIRON	10.1016/j.agee.2012.12.013
363	RAIESI F, 2012, J ARID ENVIRON	10.1016/j.jaridenv.2011.08.008
364	ZHONG ZK, 2020, PLANT SOIL	10.1007/s11104-019-04415-0
365	MEERS TL, 2008, J VEG SCI	10.3170/2008-8-18401
366	ITO J, 2016, LAND USE POLICY	10.1016/j.landusepol.2016.06.020
367	DU J, 2019, LAND-BASEL	10.3390/land8120184
368	MA WL, 2020, J AGR ECON	10.1111/1477-9552.12375

Sequence	Paper	DOI
369	KUUIRE VZ, 2016, AGR HUM VALUES	10.1007/s10460-015-9612-0
370	ZHANG Y, 2018, LAND DEGRAD DEV	10.1002/ldr.3126
371	ARMOLAITIS K, 2007, BALT FOR	NA
372	SMITH KS, 2000, ICARD 2000, VOLS I AND II, PROCEEDINGS	NA
373	LOPEZ-POMA R, 2014, INT J WILDLAND FIRE	10.1071/WF13150
374	LI WJ, 2017, LAND DEGRAD DEV	10.1002/ldr.2607
375	TAKAHASHI K, 2007, FOREST ECOL MANAG	10.1016/j.foreco.2007.05.014
376	WANG JM, 2017, ECOL ENG	10.1016/j.ecoleng.2017.02.001
377	GISPERT M, 2018, CATENA	10.1016/j.catena.2017.10.019
378	KAWADA K, 2011, GRASSL SCI	10.1111/j.1744-697X.2010.00209.x
379	KHORCHANI M, 2020, SCI TOTAL ENVIRON	10.1016/j.scitotenv.2020.137160
380	YANG Y, 2020, ONE EARTH	10.1016/j.oneear.2020.07.019
381	GU DW, 2019, LAND USE POLICY	10.1016/j.landusepol.2019.02.033
382	KUKK L, 2010, ACTA AGR SCAND B-S P	10.1080/09064710902798311
383	CASTILLO CP, 2021, ENVIRON MODELL SOFTW	10.1016/j.envsoft.2020.104946
384	RICOTTA C, 2012, NAT HAZARD EARTH SYS	10.5194/nhess-12-1333-2012
385	TULLUS T, 2013, NEW FOREST	10.1007/s11056-013-9365-9
386	LIZAGA I, 2018, LAND DEGRAD DEV	10.1002/ldr.2843
387	ZOZAYA EL, 2012, LANDSCAPE ECOL	10.1007/s10980-011-9695-y
388	YANG N, 2018, EUR J SOIL BIOL	10.1016/j.ejsobi.2018.01.003
389	ZAKKAK S, 2014, J INSECT CONSERV	10.1007/s10841-014-9663-3
390	HUTCHINSON I, 2007, RADIOCARBON	10.1017/S0033822200043198
391	WEISS J, 2005, WILD URBAN WOODLANDS: NEW PERSPECTIVES FOR URBAN FORESTRY	10.1007/3-540-26859-6_9
392	DYULGEROVA S, 2015, ACTA ZOOL BULGAR	NA
393	CAMPBELL JE, 2013, ENVIRON RES LETT	10.1088/1748-9326/8/3/035012
394	SANG N, 2014, APPL GEOGR	10.1016/j.apgeog.2014.09.015
395	LOMBA A, 2013, PLANT BIOSYST	10.1080/11263504.2012.716794
396	ZAVALLONI M, 2021, LAND USE POLICY	10.1016/j.landusepol.2019.104365
397	DE ANDRES F, 2007, SCI TOTAL ENVIRON	10.1016/j.scitotenv.2007.01.017
398	CHEN YF, 2018, J MT SCI-ENGL	10.1007/s11629-017-4452-6
399	KOZAK J, 2004, EKOL BRATISLAVA	NA
400	YU ZL, 2017, SUSTAINABILITY-BASEL	10.3390/su9020187
401	LIANG X, 2020, LAND USE POLICY	10.1016/j.landusepol.2020.104760
402	NAVAS A, 2017, LAND DEGRAD DEV	10.1002/ldr.2724
403	TULLUS A, 2012, BALT FOR	NA
404	HELM DJ, 1993, MYCORRHIZA-a	10.1007/BF00208918
405	OCHIAI A, 2001, NEW ENGL QUART	10.2307/3185461
406	NAESS JS, 2021, NAT SUSTAIN	10.1038/s41893-020-00680-5
407	ZHOU T, 2020, ENVIRON MANAGE	10.1007/s00267-020-01258-9
408	MULLER D, 2009, NATO SCI PEACE SECUR	10.1007/978-90-481-2283-7_24
409	ZHU XF, 2021, SCI TOTAL ENVIRON	10.1016/j.scitotenv.2020.142651

Sequence	Paper	DOI
410	TIAN DS, 2018, LAND DEGRAD DEV	10.1002/ldr.3137
411	SIRAMI C, 2011, BIRD STUDY	10.1080/00063657.2010.532861
412	XU HW, 2020, LAND DEGRAD DEV	10.1002/ldr.3513
413	WANG N, 2010, ENVIRON MANAGE	10.1007/s00267-010-9535-x
414	LAMERIS TK, 2016, BIODIVERS CONSERV	10.1007/s10531-015-1041-2
415	HARRIS E, 2018, SCI TOTAL ENVIRON	10.1016/j.scitotenv.2018.02.119
416	HE YF, 2020, LAND USE POLICY	10.1016/j.landusepol.2020.104826
417	KHEIRFAM H, 2020, CATENA	10.1016/j.catena.2019.104341
418	LOHMUS A, 2005, CAN J FOREST RES	10.1139/X05-047
419	DORTA-SANTOS M, 2014, SUSTAINABILITY-BASEL	10.3390/su6106902
420	XU HW, 2021, CATENA	10.1016/j.catena.2020.104812
421	PASALODOS-TATO M, 2009, SILVA FENN	10.14214/sf.176
422	SULIEMAN HM, 2009, LAND DEGRAD DEV	10.1002/ldr.894
423	HACKWORTH J, 2015, URBAN GEOGR	10.1080/02723638.2015.1011416
424	BIRD DA, 2003, ENVIRON GEOL	10.1007/s00254-003-0835-9
425	LOTTERMOSER BG, 2011, ENVIRON POLLUT	10.1016/j.envpol.2011.04.014
426	NGUYEN H, 2018, REMOTE SENS-BASEL	10.3390/rs10121973
427	LI JW, 2020, SCI TOTAL ENVIRON	10.1016/j.scitotenv.2020.140802
428	PELTZ CD, 2016, SSSA SPEC PUBL	10.2136/sssaspecpub63.2014.0047.5
429	DENG X, 2018, SUSTAINABILITY-BASEL	10.3390/su10113909
430	DENG X, 2020, LAND-BASEL	10.3390/land9100360
431	XIAO GF, 2019, J GEOGR SCI	10.1007/s11442-019-1616-z
432	CHANG EH, 2019, CATENA	10.1016/j.catena.2019.104095
433	KOLECKA N, 2019, LAND-BASEL	10.3390/land8090129
434	VOLARIK D, 2013, FOREST ECOL MANAG	10.1016/j.foreco.2012.12.016
435	CHENG LL, 2017, INT J MIN RECLAM ENV	10.1080/17480930.2016.1167305
436	KUMM KI, 2020, LAND-BASEL	10.3390/land9020042
437	SCOTT CA, 2001, BIOGEOCHEMISTRY	10.1023/A:1011877214723
438	VAROTTO M, 2014, MT RES DEV	10.1659/MRD-JOURNAL-D-14-00012.1
439	PAUDEL B, 2020, ENVIRON RES	10.1016/j.envres.2020.109711
440	TREMBLAY S, 2013, FORESTS	10.3390/f4041141
441	MIKULIC K, 2014, COMMUNITY ECOL	10.1556/COMEC.15.2014.2.5
442	DORTA-SANTOS M, 2015, ECOL ENG	10.1016/j.ecoleng.2015.03.008
443	YUSOFF NM, 2017, INT J REMOTE SENS	10.1080/01431161.2016.1266111
444	AREVALO JR, 2017, ACTA OECOL	10.1016/j.actao.2017.09.014
445	FALINSKI JB, 1980, EKOL POL-POL J ECOL	NA
446	NADAL-ROMERO E, 2011, B ASOC GEOGR ESP	NA
447	GUO YC, 2019, SUSTAINABILITY-BASEL	10.3390/su11051367
448	DENG X, 2019, INT J ENV RES PUB HE	10.3390/ijerph16193588
449	DE MIRANDA MD, 2019, SCI REP-UK	10.1038/s41598-018-38200-3
450	BAXTER RE, 2017, ANN AM ASSOC GEOGR	10.1080/24694452.2017.1298985
451	ORLOWSKI G, 2004, ACTA ORNITHOL	NA

Sequence	Paper	DOI
452	KOLECKA N, 2018, REMOTE SENS-BASEL	10.3390/rs10101568
453	YAMAGUCHI T, 2016, J LAND USE SCI	10.1080/1747423X.2016.1174317
454	MANTERO G, 2020, LANDSCAPE ECOL	10.1007/s10980-020-01147-w
455	HACKWORTH J, 2018, INT J URBAN REGIONAL	10.1111/1468-2427.12588
456	PARDINI G, 2006, AGROCHIMICA	NA
457	RAUTIAINEN A, 2016, ENVIRON SCI POLICY	10.1016/j.envsci.2015.09.011
458	ASSINI S, 2015, PLANT BIOSYST	10.1080/11263504.2014.983202
459	ERSOY A, 2008, ENVIRON TOXICOL	10.1002/tox.20314
460	WEI XC, 2011, SCI TOTAL ENVIRON	10.1016/j.scitotenv.2010.11.030
461	KUND M, 2010, EUR J FOREST RES	10.1007/s10342-010-0369-0
462	DE GOEDE RGM, 1998, APPL SOIL ECOL	10.1016/S0929-1393(98)00089-4
463	PUTTSEPP U, 2007, SILVA FENN	10.14214/sf.294
464	ARCHER N, 2002, HYDROL EARTH SYST SC	10.5194/hess-6-913-2002
465	HOUET T, 2015, J MT SCI-ENGL	10.1007/s11629-014-3404-7
466	GOGA T, 2019, REMOTE SENS-BASEL	10.3390/rs11232759
467	LASANTA T, 2020, LAND DEGRAD DEV	10.1002/ldr.3655
468	WANG JQ, 2016, J ARID LAND	10.1007/s40333-016-0042-7
469	DORMAAR JF, 1990, J RANGE MANAGE	10.2307/3898686
470	ONANS J, 1980, AUST J BOT	10.1071/BT9800479
471	LOPEZ-IGLESIAS E, 2013, RES RURAL SOCIOL DEV	10.1108/S1057-1922(2013)0000019007
472	CHAUDHARY S, 2020, LAND-BASEL	10.3390/land9030084
473	BENJAMIN K, 2008, ENVIRON MANAGE	10.1007/s00267-008-9176-5
474	LI JW, 2020, GEODERMA	10.1016/j.geoderma.2019.114167
475	DESIMINI J, 2013, J LANDSC ARCHIT	10.1080/18626033.2013.864131
476	HALL SJG, 2018, AGR SYST	10.1016/j.agsy.2018.08.009
477	HANIOKA M, 2018, AGR ECOSYST ENVIRON	10.1016/j.agee.2018.06.014
478	NAVARRO FB, 2006, LAND DEGRAD DEV	10.1002/ldr.695
479	PRISHCHEPOV AV, 2021, LAND USE POLICY	10.1016/j.landusepol.2021.105513
480	MORELL-MONZO S, 2020, REMOTE SENS-BASEL	10.3390/rs12122062
481	JOHANSSON T, 1996, NEW FOREST	NA
482	WU DM, 2019, CHEMOSPHERE	10.1016/j.chemosphere.2018.09.087
483	HOU J, 2014, CATENA	10.1016/j.catena.2013.11.009
484	PALOMEQUE X, 2017, NEW FOREST	10.1007/s11056-017-9590-8
485	BOSSO L, 2017, ACTA OECOL	10.1016/j.actao.2016.11.002
486	ZHANG K, 2012, SOIL USE MANAGE	10.1111/j.1475-2743.2012.00420.x
487	FOUCHER A, 2019, AGR ECOSYST ENVIRON	10.1016/j.agee.2019.106582
488	ZHANG LP, 2016, INT J ENV RES PUB HE	10.3390/ijerph13040354
489	SHANG ZH, 2019, CATENA	10.1016/j.catena.2018.12.002
490	KOBAYASHI Y, 2020, PLOS ONE	10.1371/journal.pone.0235846
491	RAI R, 2019, SUSTAINABILITY-BASEL	10.3390/su11195267
492	JACOB AL, 2017, RESTOR ECOL	10.1111/rec.12414
493	YANG HC, 2016, SUSTAINABILITY-BASEL	10.3390/su8111183

Sequence	Paper	DOI
494	LAUE JE, 2016, J LAND USE SCI	10.1080/1747423X.2014.993341
495	HESTERBERG D, 1993, LAND DEGRAD REHABIL	10.1002/ldr.3400040409
496	DOLTON-THORNTON N, 2021, LAND USE POLICY	10.1016/j.landusepol.2020.105269
497	BELL SM, 2021, SCI TOTAL ENVIRON	10.1016/j.scitotenv.2020.143535
498	KUNTZ KA, 2018, LAND-BASEL	10.3390/land7040128
499	PICHE N, 2015, RESTOR ECOL	10.1111/rec.12241
500	TROIANI N, 2016, ACTA BOT CROAT	10.1515/botcro-2016-0021
501	KITAZAWA M, 2019, ORNITHOL SCI	10.2326/osj.18.3
502	LOPEZ-BAO JV, 2015, BASIC APPL ECOL	10.1016/j.baae.2014.12.001
503	SROKA W, 2019, SUSTAINABILITY-BASEL	10.3390/su11113071
504	RIEDENER E, 2014, AGR ECOSYST ENVIRON	10.1016/j.agee.2013.12.023
505	UCHIDA K, 2018, J ENVIRON MANAGE	10.1016/j.jenvman.2018.04.059
506	SANCHO-REINOSO A, 2013, ERDKUNDE	10.3112/erdkunde.2013.04.01
507	GOPALAKRISHNAN T, 2020, SUSTAINABILITY-BASEL	10.3390/su12093681
508	GARCIA-BARON I, 2018, DIVERS DISTRIB	10.1111/ddi.12743
509	SUBEDI YR, 2021, ENVIRON MANAGE	10.1007/s00267-021-01461-2
510	LIU N, 2017, REMOTE SENS-BASEL	10.3390/rs9060545
511	YANG LR, 2008, ENVIRON GEOL	10.1007/s00254-007-0782-y
512	INCZE J, 2016, J MAPS	10.1080/17445647.2016.1195295
513	WEI YC, 2009, J FOREST RES-JPN	10.1007/s10310-009-0130-z
514	HELM DJ, 1993, MYCORRHIZA	10.1007/BF00208917
515	HUANG YQ, 2020, LAND-BASEL	10.3390/land9080263
516	VAN DER MERWE H, 2011, AFR J RANGE FOR SCI	10.2989/10220119.2011.642097
517	LEIRPOLL ME, 2021, RENEW ENERG	10.1016/j.renene.2020.11.159
518	YAMANAKA S, 2017, AGR ECOSYST ENVIRON	10.1016/j.agee.2017.07.027
519	YANNELLI FA, 2014, ENVIRON MANAGE	10.1007/s00267-013-0176-8
520	GRADINARU SR, 2013, PROC TECH	10.1016/j.protcy.2013.11.074
521	BIJAK S, 2014, SYLWAN	NA
522	KOLECKA N, 2021, REMOTE SENS ENVIRON	10.1016/j.rse.2021.112340
523	LASANTA T, 2019, ADV CHEM POLL ENV MG	10.1016/bs.apmp.2019.07.002
524	WILLIAMS TM, 1999, FOREST ECOL MANAG	10.1016/S0378-1127(99)00031-6
525	ZHANG FH, 2017, J AGR SCI-CAMBRIDGE	10.1017/S002185961600023X
526	CAMPOS I, 2016, COMMUNITY DEV J	10.1093/cdj/bsv051
527	SUN CL, 2018, FRONT PLANT SCI	10.3389/fpls.2018.00898
528	MASNY M, 2014, CARPATH J EARTH ENV	NA
529	WANG YW, 2020, AGR ECOSYST ENVIRON	10.1016/j.agee.2020.106821
530	MCKINSTRY MC, 1994, WETLANDS	10.1007/BF03160634
531	CAMPO J, 2019, EUR J SOIL SCI	10.1111/ejss.12799
532	HANIOKA M, 2018, BIODIVERS CONSERV	10.1007/s10531-018-1510-5
533	PENG SL, 2012, SOIL RES	10.1071/SR11149
534	AOSAAR J, 2011, BALT FOR	NA
535	LAWSON L, 2013, CITY 21ST CENTURY	NA

Sequence	Paper	DOI
536	CARVER S, 2019, ECOL REV	NA
537	SANKEY TT, 2018, LAND DEGRAD DEV	10.1002/ldr.2997
538	YUN SW, 2017, J GEOCHEM EXPLOR	10.1016/j.gexplo.2017.07.004
539	JIA YF, 2011, ARID LAND RES MANAG	10.1080/15324982.2011.554959
540	XU HW, 2020, CATENA	10.1016/j.catena.2020.104774
541	FREI T, 2020, LAND USE POLICY	10.1016/j.landusepol.2020.105034
542	WAHSHA M, 2016, J SOIL SEDIMENT	10.1007/s11368-015-1151-1
543	HEWELKE E, 2019, WATER-SUI	10.3390/w11030525
544	ROSERO-VLASOVA OA, 2018, J APPL REMOTE SENS	10.1117/1.JRS.12.042803
545	COJZER M, 2014, FORESTS	10.3390/f5112658
546	SAVULESCU I, 2019, SUSTAINABILITY-BASEL	10.3390/su11236679
547	TULLUS T, 2015, CAN J FOREST RES	10.1139/cjfr-2014-0464
548	RAJPAR H, 2019, SUSTAINABILITY-BASEL	10.3390/su11174663
549	DEGRAFF JV, 2007, REV ENG GEOL	10.1130/2007.4017(01)
550	DE BAETS S, 2012, CATENA	10.1016/j.catena.2012.01.014
551	HUHE, 2014, PLOS ONE	10.1371/journal.pone.0106714
552	NEMET E, 2016, COMMUNITY ECOL	10.1556/168.2016.17.1.10
553	LIU K, 2020, AGR ECOSYST ENVIRON	10.1016/j.agee.2019.106746
554	PIAS B, 2014, FOREST ECOL MANAG	10.1016/j.foreco.2014.06.021
555	RICCI JMP, 2018, LAND USE POLICY	10.1016/j.landusepol.2018.08.009
556	LIU M, 2021, AGR ECOSYST ENVIRON	10.1016/j.agee.2021.107629
557	SIRIN A, 2018, LAND-BASEL	10.3390/land7020071
558	CHENG LL, 2019, INT J COAL SCI TECHN	10.1007/s40789-019-0241-x
559	PELLIS G, 2019, SCI TOTAL ENVIRON	10.1016/j.scitotenv.2019.03.329
560	GOMES ACS, 2012, RESTOR ECOL	10.1111/j.1526-100X.2011.00773.x
561	HOU DW, 2021, LANDSCAPE URBAN PLAN	10.1016/j.lurbplan.2021.104170
562	RADOVIC AA, 2013, FOLIA ZOOL	NA
563	LU C, 2020, GROWTH CHANGE	10.1111/grow.12431
564	JANUS J, 2019, J LAND USE SCI	10.1080/1747423X.2019.1709226
565	HAN Z, 2020, APPL GEOGR	10.1016/j.apgeog.2020.102245
566	GRADINARU SR, 2020, CARPATH J EARTH ENV	10.26471/cjees/2020/015/119
567	HUHE, 2016, PLOS ONE	10.1371/journal.pone.0154697
568	CHENG WF, 2011, SPECTROSC SPECT ANAL	10.3964/j.issn.1000-0593(2011)06-1615-06
569	BAI WJ, 2010, ARID LAND RES MANAG	10.1080/15324981003635461
570	QIU BW, 2020, LAND DEGRAD DEV	10.1002/ldr.3617
571	MACCHERINI S, 2013, BIODIVERS CONSERV	10.1007/s10531-013-0571-8
572	YOSHIHARA Y, 2009, J ARID ENVIRON	10.1016/j.jaridenv.2008.09.017
573	KEPFER-ROJAS S, 2015, APPL VEG SCI	10.1111/avsc.12169
574	THOGMARTIN WE, 2009, RESTOR ECOL	10.1111/j.1526-100X.2007.00344.x
575	HEYDARI M, 2020, ECOL ENG	10.1016/j.ecoleng.2020.105963
576	LIU HF, 2019, LAND DEGRAD DEV	10.1002/ldr.3372
577	SILVESTRINI M, 2012, REV ARVORE	10.1590/S0100-67622012000400008

Sequence	Paper	DOI
578	MAHANEY WM, 2010, PLANT SOIL	10.1007/s11104-009-0178-8
579	YOSHIKAWA S, 2004, JARQ-JPN AGR RES Q	10.6090/jarq.38.21
580	SON Y, 2006, J PLANT BIOL	10.1007/BF03030538
581	DING Y, 2005, J INTEGR PLANT BIOL	10.1111/j.1744-7909.2005.00026.x
582	JANOVSKY MP, 2020, CATENA	10.1016/j.catena.2019.104347
583	KING MA, 1989, J RANGE MANAGE	10.2307/3899468
584	HAUCHHUM R, 2019, NUTR CYCL AGROECOSYS	10.1007/s10705-019-09972-5
585	HEIDER K, 2021, REG ENVIRON CHANGE	10.1007/s10113-020-01739-x
586	ZENG LZ, 1993, AUST GEOGR	NA
587	ZAKKAK S, 2018, J VEG SCI	10.1111/jvs.12670
588	ZHANG Z, 2017, J SOIL SEDIMENT	10.1007/s11368-017-1650-3
589	GARDINER DT, 1993, ARID SOIL RES REHAB	10.1080/15324989309381337
590	SEDLAR Z, 2018, PLANT BIOSYST	10.1080/11263504.2017.1330774
591	SHIN MW, 2020, SUSTAINABILITY-BASEL	10.3390/su12010334
592	CHAUDHARY S, 2020, LAND-BASEL-a	10.3390/land9010001
593	ROBLES AB, 2002, J ARID ENVIRON	10.1006/jare.2001.0913
594	KOLECKA N, 2016, INT ARCH PHOTOGRAMM	10.5194/isprsarchives-XLI-B8-931-2016
595	LEMIEUX C, 1998, PHYTOPROTECTION	10.7202/706132ar
596	RODRIGUEZ-FRANCO C, 2021, BIOCHAR	10.1007/s42773-020-00074-y
597	GOPALAKRISHNAN T, 2021, AGRICULTURE-BASEL	10.3390/agriculture11030211
598	WANG HL, 2020, LAND DEGRAD DEV	10.1002/ldr.3523
599	WANG JS, 2013, EUR J FOREST RES	10.1007/s10342-012-0676-8
600	DING Y, 2006, J INTEGR PLANT BIOL	10.1111/j.1744-7909.2006.00262.x
601	WANG HL, 2019, APPL MICROBIOL BIOT	10.1007/s00253-018-09575-0
602	LI JJ, 2011, SOIL SCI	10.1097/SS.0b013e31821d6e7c
603	TABENI S, 2016, ECOL INDIC	10.1016/j.ecolind.2016.02.019
604	VAZ AS, 2019, LANDSCAPE URBAN PLAN	10.1016/j.landurbplan.2019.01.009
605	GOMORY D, 2006, POL J ECOL	NA
606	GISI U, 1981, ACTA OECOL-OEC PLANT-a-b	NA
607	TAROLLI P, 2019, ENVIRON HIST-SER	10.1007/978-3-319-96815-5_12
608	KOBAYASHI Y, 2018, ECOL RES MONOGR-a	10.1007/978-981-10-7203-1_13
609	ZGLOBICKI W, 2020, APPL SCI-BASEL	10.3390/app10103500
610	DUBOVYK O, 2012, INT GEOSCI REMOTE SE	10.1109/IGARSS.2012.6352089
611	ZAKKAK S, 2015, AMPHIBIA-REPTILIA	10.1163/15685381-00003002
612	NEKHAY O, 2009, SPAN J AGRIC RES	NA
613	ZHAO YG, 2019, ARCH MICROBIOL	10.1007/s00203-019-01689-x
614	REBELE F, 2014, TUEXENIA	NA
615	ESTRUCH C, 2018, PLANT SOIL	10.1007/s11104-018-3581-1
616	MIDRIAK R, 1995, EKOL BRATISLAVA	NA
617	AN Y, 2019, COMMUNITY ECOL	10.1556/168.2019.20.1.3
618	WANG R, 2019, LAND-BASEL	10.3390/land8120186
619	ZAMBON I, 2018, SUSTAINABILITY-BASEL	10.3390/su10041159

Sequence	Paper	DOI
620	DAX T, 2021, LAND-BASEL	10.3390/land10060591
621	SACKOV I, 2020, REMOTE SENS-BASEL	10.3390/rs12244189
622	BARRENA-GONZALEZ J, 2020, CENT EURO FOR J	10.2478/forj-2020-0015
623	SCHERER G, 2021, INSECT CONSERV DIVER	10.1111/icad.12485
624	HORASAN BY, 2020, ENVIRON EARTH SCI	10.1007/s12665-020-08985-6
625	MIHALIK J, 2011, WATER AIR SOIL POLL	10.1007/s11270-010-0518-6
626	MINOTTA G, 2011, IFOREST	10.3832/ifor0560-004
627	LIU XH, 2020, SCI TOTAL ENVIRON	10.1016/j.scitotenv.2020.137148
628	MEDVEDEVA MA, 2017, IZV ATMOS OCEAN PHY+	10.1134/S0001433817090201
629	LIU XZ, 2011, AFR J BIOTECHNOL	NA
630	KHANINA LG, 2018, ECOL PROCESS	10.1186/s13717-018-0150-8
631	GISI U, 1981, ACTA OECOL-OEC PLANT-a-b-c	NA
632	SONG W, 2019, SUSTAINABILITY-BASEL	10.3390/su11215951
633	YANG JD, 2019, ECOL ENG	10.1016/j.ecoleng.2019.04.026
634	WANG YH, 2020, SUSTAINABILITY-BASEL	10.3390/su12083356
635	BISTA R, 2021, EARTH INTERACT	10.1175/EI-D-21-0006.1
636	SZOSTAK M, 2018, GEOD CARTOGR	10.24425/gac.2018.125476
637	LEE J, 2021, LAND USE POLICY	10.1016/j.landusepol.2021.105544
638	PECO JD, 2021, SUSTAINABILITY-BASEL	10.3390/su13126555
639	JUAN JER, 2016, B ASOC GEOGR ESP	10.21138/bage.2272
640	TEREKHIN EA, 2017, COMPUT OPT	10.18287/2412-6179-2017-41-5-719-725
641	OLSEN VM, 2021, NAT FOOD	10.1038/s43016-021-00417-3
642	O'ROURKE E, 2019, EUR COUNTRYS	10.2478/euco-2019-0011
643	LI J, 2018, ENVIRON EARTH SCI	10.1007/s12665-018-7385-7
644	WANG R, 2020, SCI TOTAL ENVIRON	10.1016/j.scitotenv.2020.141416
645	RUIZ-LENDINEZ JJ, 2020, ISPRS INT J GEO-INF	10.3390/ijgi9040191
646	ARMOLAITIS K, 2011, ZEMDIRBYSTE	NA
647	DENG N, 2019, FRONT MICROBIOL	10.3389/fmicb.2019.00262
648	LAN JC, 2020, ECOL ENG	10.1016/j.ecoleng.2020.106033
649	OHASHI H, 2016, ECOL EVOL	10.1002/ece3.2514
650	ZHANG LP, 2018, ISPRS INT J GEO-INF	10.3390/ijgi7080305
651	ZARAGOZI BM, 2011, SEARCH I SER DEV ATT	10.2495/ECO110321
652	ZHANG C, 2012, ADV MATER RES-SWITZ	10.4028/www.scientific.net/AMR.356-360.2422
653	ASLAN A, 2021, SCI TOTAL ENVIRON	10.1016/j.scitotenv.2021.148320
654	HAUCHHUM R, 2020, TROP ECOL	10.1007/s42965-020-00079-5
655	CHAMBERS JC, 2014, INVAS PLANT SCI MANA	10.1614/IPSM-D-13-00046.1
656	PRAGYA N, 2017, GLOB ECOL CONSERV	10.1016/j.gecco.2017.06.002
657	YANG L, 2020, ECOL EVOL	10.1002/ece3.6067
658	TOMUSIAK R, 2014, SYLWAN	NA
659	BARDULIS A, 2012, RES RURAL DEV	NA
660	PISABARRO A, 2019, GEOGR ANN A	10.1080/04353676.2019.1591042
661	MORELL-MONZO S, 2021, REMOTE SENS-BASEL	10.3390/rs13040681

Sequence	Paper	DOI
662	ZHANG SW, 2018, SCI REP-UK	10.1038/s41598-018-35624-9
663	DAVIS JK, 2008, AFR J RANGE FOR SCI	10.2989/AJRFS.2008.25.2.3.482
664	FEDRIGOTTI C, 2016, GLOBAL NEST J	NA
665	RYTTER RM, 2018, IFOREST	10.3832/ifor2853-011
666	WOLINSKA A, 2020, AGRONOMY-BASEL	10.3390/agronomy10111795
667	SHOYAMA K, 2021, ENVIRON MANAGE	10.1007/s00267-021-01497-4
668	SAKELLARIOU M, 2021, LAND-BASEL	10.3390/land10050457
669	LI H, 2021, LAND-BASEL	10.3390/land10111206
670	YOON H, 2020, ISPRS J PHOTOGRAMM	10.1016/j.isprsjprs.2020.05.021
671	LI WJ, 2012, 2012 5TH INTERNATIONAL CONGRESS ON IMAGE AND SIGNAL PROCESSING (CISP)	NA
672	CASTRO P, 2019, SPRINGER SER ENV MAN	10.1007/978-3-319-89644-1_6
673	HANOUSKOVA I, 1999, PERSPECTIVES IN ECOLOGY: A GLANCE FROM THE VII INTERNATIONAL CONGRESS OF ECOLOGY	NA
674	WANG SX, 2013, ADV INTEL SYS RES	NA
675	DENG X, 2021, J LAND USE SCI	10.1080/1747423X.2021.1954707
676	ESTOQUE RC, 2019, SCI TOTAL ENVIRON	10.1016/j.scitotenv.2019.07.204
677	CZESAK B, 2021, REMOTE SENS-BASEL	10.3390/rs13061166
678	LI YL, 2021, GEOGR SUSTAIN	10.1016/j.geosus.2021.06.003
679	XIAO L, 2021, CATENA	10.1016/j.catena.2020.105081
680	KRYSIAK S, 2020, LAND-BASEL	10.3390/land9030082
681	SULLIVAN B, 2016, TRANSPORT RES REC	10.3141/2580-09
682	LIU WC, 2020, J ARID LAND	10.1007/s40333-020-0009-6
683	LASANTA T, 2021, CUAD INVESTIG GEOGR	10.18172/cig.4755
684	ZAMPELLA RA, 2008, WETLANDS	10.1672/07-116.1
685	DESYATKIN AR, 2018, ATMOSPHERE-BASEL	10.3390/atmos9080308
686	TRAPPE J, 2017, J INSECT CONSERV	10.1007/s10841-017-0021-0
687	ZITTI M, 2018, INT J ECOL DEV	NA
688	LI DD, 2021, APPL SOIL ECOL	10.1016/j.apsoil.2020.103808
689	HOU J, 2014, ENVIRON MONIT ASSESS	10.1007/s10661-013-3444-4
690	KHORCHANI M, 2021, CATENA	10.1016/j.catena.2021.105374
691	XYSTRAKIS F, 2014, ACTA BOT CROAT	10.2478/botcro-2013-0021
692	LEE S, 2020, GISCI REMOTE SENS	10.1080/15481603.2019.1698490
693	TAO MX, 2018, FRESEN ENVIRON BULL	NA
694	NADAL-ROMERO E, 2021, CATENA	10.1016/j.catena.2021.105441
695	ABADIE J, 2021, J VEG SCI	10.1111/jvs.12860
696	KUZNETSOVA T, 2009, BALT FOR	NA
697	SHI CD, 2018, ENRGY PROCED	10.1016/j.egypro.2018.10.041
698	JIM CY, 1997, GEOGRAPHY	NA
699	AMADO TJC, 2020, BURL DODDS AGR SCI	10.19103/AS.2019.0049.14
700	SHIRAZI MA, 1984, ENVIRON MANAGE	10.1007/BF01868027
701	RUSSELL WB, 1985, CAN FIELD NAT	NA

Sequence	Paper	DOI
702	CHIDI CL, 2017, SPRING GEOGR	10.1007/978-981-10-2890-8_6
703	TSUTSUMI M, 2012, GRASS FORAGE SCI	10.1111/j.1365-2494.2011.00819.x
704	SIMMONS SA, 2002, NORTHWEST SCI	NA
705	TSUTSUMI M, 2014, GRASSL SCI	10.1111/grs.12054
706	SASAKI K, 2021, LAND-BASEL	10.3390/land10101031
707	CHAUDHARY S, 2019, SUSTAINABILITY-BASEL	10.3390/su11246931
708	PURGER JJ, 2011, AVIAN BIOL RES	10.3184/175815511X13000366671256
709	ZARAGOZI B, 2013, WIT TRANS ECOL ENVIR	10.2495/ECO130191
710	SU GD, 2020, CHINESE GEOGR SCI	10.1007/s11769-020-1146-8
711	ZHANG C, 2012, SOIL SCI	10.1097/SS.0b013e318270a637
712	MALECKA M, 2014, SYLWAN	NA
713	RUSINA S, 2021, NEW FOREST	10.1007/s11056-020-09809-y
714	COJZER M, 2010, SUMAR LIST	NA
715	HU A, 2021, LAND DEGRAD DEV	10.1002/ldr.3780
716	WEILER A, 2013, RESTOR ECOL	10.1111/rec.12006
717	WU MH, 2020, REMOTE SENS APPL	10.1016/j.rsase.2020.100403
718	LOUPASAKIS C, 2008, Q J ENG GEOL HYDROGE	10.1144/1470-9236/07-037
719	WEI ZH, 2021, REMOTE SENS-BASEL	10.3390/rs13132549
720	YUN SW, 2020, J CHEM-NY	10.1155/2020/9671871
721	COSSART E, 2020, SUSTAINABILITY-BASEL	10.3390/su12114695
722	TUMELIENE E, 2021, SUSTAINABILITY-BASEL	10.3390/su13126941
723	WANG RJ, 2021, J ENVIRON MANAGE	10.1016/j.jenvman.2021.112227
724	KUKULS I, 2015, NORDIC VIEW TO SUSTAINABLE RURAL DEVELOPMENT	NA
725	KIM KH, 1996, J ENVIRON SCI HEAL A	10.1080/10934529609376388
726	SULIEMAN HM, 2014, AFR J ECOL	10.1111/aje.12108
727	AYE WM, 2020, PADDY WATER ENVIRON	10.1007/s10333-020-00791-x
728	LOZADA JR, 2006, INTERCIENCIA	NA
729	CHANG J, 2015, LEGISLATION, TECHNOLOGY AND PRACTICE OF MINE LAND RECLAMATION	NA
730	SREEDHAR Y, 2017, FRESEN ENVIRON BULL	NA
731	SCHMITZ MF, 2021, LAND-BASEL	10.3390/land10070721
732	CAO JJ, 2020, LAND USE POLICY	10.1016/j.landusepol.2020.105027
733	VIMIC AV, 2021, ATMOSPHERE-BASEL	10.3390/atmos12081054
734	SULEVMANOV R, 2020, SPAN J SOIL SCI	10.3232/SJSS.2020.V10.N1.03
735	JANUS J, 2021, ECOL INDIC	10.1016/j.ecolind.2020.106904
736	PALMERO-INIESTA M, 2021, LAND-BASEL	10.3390/land10080817
737	TRIANTAFYLLIDIS S, 2020, ENVIRON EARTH SCI	10.1007/s12665-020-09265-z
738	TARNAWCZYK M, 2021, MINERALS-BASEL	10.3390/min11060559
739	BOBROVSKY MV, 2018, KNE LIFE SCI	10.18502/kls.v4i7.3222
740	NELSON M, 1990, LANDSCAPE URBAN PLAN	10.1016/0169-2046(90)90009-Q
741	CALL CA, 1986, SOUTHWEST NAT	10.2307/3671841

Sequence	Paper	DOI
742	GISI U, 1981, ACTA OECOL-OEC PLANT	NA
743	GISI U, 1981, ACTA OECOL-OEC PLANT-a	NA
744	WIEGER A, 1982, GEOGR Z	NA
745	BUCHA T, 2021, REMOTE SENS-BASEL	10.3390/rs13132488
746	LI YM, 2021, INT J ENV RES PUB HE	10.3390/ijerph18041815
747	NIE L, 2019, INT J SYST EVOL MICR	10.1099/ijsem.0.003578
748	PEREZ-LUQUE AJ, 2021, FORESTS	10.3390/f12111584
749	SZATMARI D, 2018, INT CONF CARTOGR GIS	NA
750	SOYDAN H, 2015, INT GEOSCI REMOTE SE	NA
751	LAZDINS A, 2011, RURAL DEVELOPMENT	NA
752	WANG YW, 2020, ACTA GEOCHIM	10.1007/s11631-019-00389-z
753	KHORCHANI M, 2021, HYDROL PROCESS	10.1002/hyp.14191
754	WANG YW, 2021, LAND-BASEL	10.3390/land10121341
755	COJZER M, 2019, ACTA SILVAE LIGNI	10.20315/ASetL.119.3
756	KATAYAMA N, 2021, AGR ECOSYST ENVIRON	10.1016/j.agee.2021.107539
757	LIU J, 2012, ADV MATER RES-SWITZ	10.4028/www.scientific.net/AMR.356-360.726
758	TROYAN J, 2019, ECONOMIES	10.3390/economies7010003
759	ZHANG ZG, 2020, PEERJ	10.7717/peerj.10349
760	OKSUZ DP, 2021, AGROFOREST SYST	10.1007/s10457-021-00649-z
761	TAKAYAMA T, 2021, AUST J AGR RESOUR EC	10.1111/1467-8489.12425
762	STEPHENSON HG, 1996, CIM BULL	NA
763	MOVAHEDI R, 2021, LAND USE POLICY	10.1016/j.landusepol.2021.105588
764	DE BEURS KM, 2004, INT GEOSCI REMOTE SE	NA
765	RONG GH, 2021, AGR ECOSYST ENVIRON	10.1016/j.agee.2021.107505
766	DRAMSTAD WE, 2021, LAND-BASEL	10.3390/land10111136
767	REGA C, 2020, CITIES NATURE	10.1007/978-3-030-33027-9_5
768	SEO J, 2020, ECON ENVIRON GEOL	10.9719/EEG.2020.53.6.743
769	DELCROS P, 2005, REV GEOGR ALP	10.3406/rga.2005.2358
770	SCORER C, 2019, S AFR GEOGR J	10.1080/03736245.2018.1541018
771	SARES MA, 2000, ICARD 2000, VOLS I AND II, PROCEEDINGS	NA
772	TOBRATOV SA, 2019, AMAZON INVESTIG	NA
773	FRADETTE O, 2021, FOREST ECOL MANAG	10.1016/j.foreco.2021.119565
774	SANTARSIERO V, 2021, LECT NOTES COMPUT SC	10.1007/978-3-030-86979-3_49
775	SCOTT CA, 2001, BIOGEOCHEMISTRY-a	10.1023/A:1011840116540
776	CASERTA G, 2000, PERIOD BIOL	NA
777	TEREKHIN EA, 2021, COMPUT OPT	10.18287/2412-6179-CO-797
778	HOLDEN PB, 2021, LAND USE POLICY	10.1016/j.landusepol.2021.105429
779	MIYAKE S, 2016, PAPERS OF THE 24TH EUROPEAN BIOMASS CONFERENCE: SETTING THE COURSE FOR A BIOBASED ECONOMY	NA
780	MORENO-RODRIGUEZ V, 2020, J S AM EARTH SCI	10.1016/j.jsames.2020.102829
781	WANG Y, 2016, SPRINGERPLUS	10.1186/s40064-016-2678-3

Sequence	Paper	DOI
782	TANGHE M, 1984, B SOC ROY BOT BELG	NA
783	SCHUMAN GE, 1999, REMEDIATION AND MANAGEMENT OF DEGRADED LANDS	NA
784	SCHREIBER KF, 1981, ANGEW BOT	NA
785	TAN SK, 2004, MANAGEMENT SCIENCES AND GLOBAL STRATEGIES IN THE 21ST CENTURY, VOLS 1 AND 2	NA
786	LI GY, 2021, LAND-BASEL	10.3390/land10101049
787	CHENG Q, 2013, ADV EDUC RES	NA
788	BENISTON JW, 2017, ENCYCLOPEDIA OF SOIL SCIENCE, VOLS I-III, 3RD EDITION	10.1081/E-ESS3-120053535
789	GRUIZ K, 2014, ENGINEERING TOOLS FOR ENVIRONMENTAL RISK MANAGEMENT - I: ENVIRONMENTAL DETERIORATION AND CONTAMINATION - PROBLEMS AND THEIR MANAGEMENT	NA
790	YAMANAKA S, 2018, ECOL RES MONOGR	10.1007/978-981-10-7203-1_15
791	LIESKOVSKY J, 2021, LAND-BASEL	10.3390/land10040334
792	KITANO S, 2021, LAND-BASEL	10.3390/land10060596
793	YANG L, 2021, ZOOL STUD	10.6620/ZS.2021.60-72
794	KITAZAWA M, 2021, BIODIVERS CONSERV	10.1007/s10531-021-02178-8
795	AFOLAYAN AJ, 1990, REV BIOL TROP	NA
796	SPIRA Y, 2014, ENGINEERING TOOLS FOR ENVIRONMENTAL RISK MANAGEMENT - I: ENVIRONMENTAL DETERIORATION AND CONTAMINATION - PROBLEMS AND THEIR MANAGEMENT	NA
797	LIU QS, 2015, 2015 8TH INTERNATIONAL CONGRESS ON IMAGE AND SIGNAL PROCESSING (CISP)	NA
798	KOBAYASHI Y, 2018, ECOL RES MONOGR	10.1007/978-981-10-7203-1_17
799	MESCH M, 2001, BAT CONSERVATION AND MINING: A TECHNICAL INTERACTIVE FORUM, PROCEEDINGS	NA
800	CASTRO P, 2020, LAND USE POLICY	10.1016/j.landusepol.2020.104633
801	ZARAGOZI B, 2020, DATA BRIEF	10.1016/j.dib.2020.106340
802	ZHAO LY, 2021, REMOTE SENS-BASEL	10.3390/rs13204057
803	ZHAO CZ, 2010, 2010 INTERNATIONAL CONFERENCE AGRICULTURAL SCIENCES AND ENGINEERING (CASE 2010)	NA
804	SHEA P, 2000, ICARD 2000, VOLS I AND II, PROCEEDINGS	NA
805	VOLKOVA I, 2020, J FOR SCI-PRAGUE	10.17221/100/2020-JFS
806	CHEN DL, 2021, SCI TOTAL ENVIRON	10.1016/j.scitotenv.2021.148155
807	SZATMARI D, 2021, CAN J REMOTE SENS	10.1080/07038992.2021.1929118
808	PURWAWANGSA H, 2021, J MANAJ HUTAN TROPIK	10.7226/jtfm.27.te.32
809	GAYLEY K, 2020, TAIWANIA	10.6165/tai.2020.65.336
810	NIZKIY S, 2020, E3S WEB CONF	10.1051/e3sconf/202020302005

Sequence	Paper	DOI
811	ZHU QW, 2017, LAND RECLAMATION IN ECOLOGICAL FRAGILE AREAS	NA
812	AHMED A, 2021, LAND USE POLICY	10.1016/j.landusepol.2021.105492
813	LEE H, 2020, ECON ENVIRON GEOL	10.9719/EEG.2020.53.2.133
814	KARMINI, 2020, FOR SOC	10.24259/fs.v4i1.8939
815	GALLAGHER D, 1999, ENVIRONMENTAL ENGINEERING 1999	NA
816	FUJII Y, 2021, INT J HOUS MARK ANAL	10.1108/IJHMA-05-2020-0054
817	TAN Y, 2014, APPL MECH MATER	10.4028/www.scientific.net/AMM.448-453.923
818	ZHANG LP, 2017, LAND RECLAMATION IN ECOLOGICAL FRAGILE AREAS	NA
819	MATSUSHIMA N, 2021, OECOLOGIA	10.1007/s00442-021-04991-y
820	REBOLA LC, 2021, SCI TOTAL ENVIRON	10.1016/j.scitotenv.2021.149487
821	COGLIASTRO A, 2019, FOREST CHRON	10.5558/tfc2019-014
822	PANG W, 2019, APPL ECOL ENV RES	10.15666/aeer/1702_22332247
823	GAUDESIUS R, 2017, ENVIRON ENG-VILNIUS	10.3846/enviro.2017.190
824	WANG LD, 2021, J ENVIRON PROT ECOL	NA
825	ZHANG JF, 2020, STUDY OF ECOLOGICAL ENGINEERING OF HUMAN SETTLEMENTS	10.1007/978-981-15-1373-2_6
826	SMITH BD, 2000, ICARD 2000, VOLS I AND II, PROCEEDINGS	NA
827	SCIUBBA L, 2021, AGRONOMY-BASEL	10.3390/agronomy11091841
828	GU MH, 2021, FRONT ENV SCI-SWITZ	10.3389/fenvs.2021.580775
829	QUIJADA LVC, 2021, HIST AGRAR	10.26882/histagrar.083e01C
830	LEIVA CL, 2020, DOC ANAL GEOGR	10.5565/rev/dag.621
831	SZYMANIUK Z, 2018, PROCEEDINGS OF THE 2018 INTERNATIONAL SCIENTIFIC CONFERENCE - ECONOMIC SCIENCES FOR AGRIBUSINESS AND RURAL ECONOMY, NO 2, 2018	10.22630/ESARE.2018.2.11
832	ESTEVEZ E, 2021, FRONT WATER	10.3389/frwa.2021.682608
833	CRAMER AS, 2021, MINERALS-BASEL	10.3390/min11040365
834	LI XD, 2017, LAND RECLAMATION IN ECOLOGICAL FRAGILE AREAS	NA
835	BAEVA YI, 2019, SPRING GEOGR	10.1007/978-3-319-89602-1_12
836	KIRKBY M, 2005, IAHS-AISH P	NA
837	SKOKANOVA H, 2016, CENTRAL EUROPE AREA IN VIEW OF CURRENT GEOGRAPHY	NA
838	DIAO HJ, 2021, J PLANT ECOL	10.1093/jpe/rtab006
839	FRAIDE EMC, 2018, ESTUD DERECHO	10.17533/udea.esde.v75n166a04
840	XU DY, 2013, ADV MATER RES-SWITZ	10.4028/www.scientific.net/AMR.726-731.4976
841	BALAMI S, 2021, FOREST ECOL MANAG	10.1016/j.foreco.2021.119181
842	GAO X, 2021, PLOS ONE	10.1371/journal.pone.0255509

Sequence	Paper	DOI
843	SARES MA, 1996, PROCEEDINGS OF THE SYMPOSIUM ON THE APPLICATION OF GEOPHYSICS TO ENGINEERING AND ENVIRONMENTAL PROBLEMS	NA
844	HE MX, 2014, APPL MECH MATER	10.4028/www.scientific.net/AMM.448-453.978
845	ZHANG FH, 2007, PRINCIPLES AND PRACTICES OF DESERTIFICATION CONTROL, VOL I	NA
846	KOLODNY O, 2016, J CONCHOL	NA
847	KARA F, 2021, ENVIRON MONIT ASSESS	10.1007/s10661-021-09403-5
848	CRAVOTTA CA, 2017, ENVIRON ENG GEOSCI	10.2113/gseegeosci.23.4.243
849	KOURKOUMPAS DS, 2018, PAPERS OF THE 26TH EUROPEAN BIOMASS CONFERENCE: SETTING THE COURSE FOR A BIOBASED ECONOMY	NA
850	KAKEMBO V, 2019, CLIM CHANG MANAG	10.1007/978-3-030-12974-3_14
851	GUO FX, 2021, FORESTS	10.3390/f12091228
852	ABELLA SR, 2021, APPL VEG SCI	10.1111/avsc.12629
853	HE S, 2021, REMOTE SENS-BASEL	10.3390/rs13193956
854	ZHUANG CL, 2005, LECT NOTES ARTIF INT	NA
855	DOLNEY TJ, 2013, EMERGING METHODS AND MULTIDISCIPLINARY APPLICATIONS IN GEOSPATIAL RESEARCH	10.4018/978-1-4666-1951-7.ch010
856	DOLNEY TJ, 2013, GEOGRAPHIC INFORMATION SYSTEMS: CONCEPTS, METHODOLOGIES, TOOLS, AND APPLICATIONS, VOL 1	10.4018/978-1-4666-2038-4.ch126
857	SALAMON JA, 2020, ECOL EVOL	10.1002/ece3.6535
858	CHAMID C, 2020, IOP CONF SER-MAT SCI	10.1088/1757-899X/830/3/032070
859	ALAOUI ML, 2005, ACTA BOT GALLICA	10.1080/12538078.2005.10515506
860	GARTSHORE H, 2019, LABOUR HIST-AUST	10.3828/jlh.2019.21
861	GAWEDA T, 2021, FORESTS	10.3390/f12070956
862	SULIEMAN HM, 2007, 2007 INTERNATIONAL WORKSHOP ON THE ANALYSIS OF MULTI-TEMPORAL REMOTE SENSING IMAGES	NA
863	YANG F, 2021, ARCH AGRON SOIL SCI	10.1080/03650340.2020.1820487
864	NORDEN B, 2021, FOREST ECOL MANAG	10.1016/j.foreco.2021.119045
865	MAKTAV D, 1996, EARTH OBS REMOT SEN+	NA
866	SHI CD, 2019, IOP C SER EARTH ENV	10.1088/1755-1315/267/6/062006
867	NABYTOVYC I, 2021, KJNIZEV SMOTRA	NA
868	MICHAELS PJ, 1985, CLIMATIC CHANGE	10.1007/BF00140505
869	MATHISEN E, 2018, CIV WAR AM	NA
870	HOLMBERG GV, 1980, T ASAE	NA
871	RICHMOND TC, 1995, SUDBURY '95 - MINING AND THE ENVIRONMENT, CONFERENCE PROCEEDINGS, VOLS 1-3	NA
872	ROBLIN KE, 2007, OIL GAS J	NA

Sequence	Paper	DOI
873	TAN YR, 1990, SOIL CROP SCI SOC FL	NA
874	WORKMAN JL, 1991, PROCEEDINGS OF THE SEVENTEENTH ANNUAL CONFERENCE ON EXPLOSIVES AND BLASTING TECHNIQUE, VOL 2	NA
875	ROBINSON NA, 1990, PROCEEDINGS OF THE 1990 MINING AND RECLAMATION CONFERENCE AND EXHIBITION, VOLS 1 AND 2	NA
876	DATTAVIO LE, 1980, J ENVIRON QUAL	10.2134/jeq1980.00472425000900040014x
877	ZAUSKOVA L, 2013, HIST CASOPIS	NA
878	COMBE MM, 2018, EDINB LAW REV	10.3366/elr.2018.0490
879	BORGER I, 1999, ARCHIT DIGEST	NA
880	CALDWELL C, 1992, ACHIEVING LAND USE POTENTIAL THROUGH RECLAMATION	NA
881	CURULLI I, 2007, PLACES-FORUM ENVIRON	NA
882	HUTCHINGS MJ, 1989, BRIGHTON CROP PROTECTION CONFERENCE - WEEDS 1989, VOLS 1-3	NA
883	ASTON RL, 2001, MINER RESOUR ENG	NA
884	IANNACCHIONE AT, 1995, ROCK MECHANICS - PROCEEDINGS OF THE 35TH U.S. SYMPOSIUM	NA
885	DARRACQ S, 1995, LANDSCAPE ECOLOGY IN LAND USE PLANNING METHODS AND PRACTICE	NA
886	HUTNIK RJ, 1990, PROCEEDINGS OF THE 1990 MINING AND RECLAMATION CONFERENCE AND EXHIBITION, VOLS 1 AND 2	NA
887	SANTOPIETRO GD, 1996, J SOIL WATER CONSERV	NA
888	RUSSELL LJ, 1990, PROCEEDINGS OF THE FOURTH WESTERN REGIONAL CONFERENCE ON PRECIOUS METALS AND THE ENVIRONMENT	NA
889	SOPPER WE, 1990, PROCEEDINGS OF THE 1990 MINING AND RECLAMATION CONFERENCE AND EXHIBITION, VOLS 1 AND 2	NA
890	RUSSO RO, 1995, NITR FIX TREE RES	NA
891	MICHAUD LH, 1995, SUDBURY '95 - MINING AND THE ENVIRONMENT, CONFERENCE PROCEEDINGS, VOLS 1-3	NA
892	MESCH M, 2001, BAT CONSERVATION AND MINING: A TECHNICAL INTERACTIVE FORUM, PROCEEDINGS-a	NA
893	OHARA KC, 1992, PROCEEDINGS OF THE 43RD ANNUAL HIGHWAY GEOLOGY SYMPOSIUM	NA
894	STANDFIELD R, 2012, EMPIRE PERSPECT	10.5937/rudrad1201061S
895	ZIPPER CE, 1992, ACHIEVING LAND USE POTENTIAL THROUGH RECLAMATION	NA
896	ROBBIE J, 2021, EDINB LAW REV	10.3366/elr.2021.0716

Supplementary Table S2 Most Global Cited Documents 100 (theme)

Paper	DOI	Total Citations	Main research	Appears as a marginal study (appears as a sub study of other studies, or is only mentioned)	The study was not mentioned (the study on abandonment of farmland was not carried out)
FARGIONE J, 2008, SCIENCE	10.1126/science.1152747	2450		√	
MACDONALD D, 2000, J ENVIRON MANAGE	10.1006/jema.1999.0335	1285	√		
RAMANKUTTY N, 1999, GLOBAL BIOGEOCHEM CY	10.1029/1999GB900046	1281		√	
HOBBS RJ, 2006, GLOBAL ECOL BIOGEOGR	10.1111/j.1466-822x.2006.00212.x	1184			√
PONTIUS RG, 2011, INT J REMOTE SENS	10.1080/01431161.2011.552923	1058			√
VONUEXKULL HR, 1995, PLANT SOIL	10.1007/BF00009558	998			√
SCHIMEL DS, 2001, NATURE	10.1038/35102500	895		√	
LAMB D, 2005, SCIENCE	10.1126/science.1111773	841			√
STOATE C, 2001, J ENVIRON MANAGE	10.1006/jema.2001.0473	811		√	
GUARIGUATA MR, 2001, FOREST ECOL MANAG	10.1016/S0378-1127(00)00535-1	809			√
STOATE C, 2009, J ENVIRON MANAGE	10.1016/j.jenvman.2009.07.005	782		√	
HOUGHTON RA, 1999, SCIENCE	10.1126/science.285.5427.574	750		√	

Paper	DOI	Total Citations	Main research	Appears as a marginal study (appears as a sub study of other studies, or is only mentioned)	The study was not mentioned (the study on abandonment of farmland was not carried out)
KEESSTRA SD, 2016, SOIL-GERMANY	10.5194/soil-2-111-2016	675			√
BAKKER JP, 1999, TRENDS ECOL EVOL	10.1016/S0169-5347(98)01544-4	639			√
CHAZDON RL, 2003, PERSPECT PLANT ECOL	10.1078/1433-8319-00042	635			√
GOODALE CL, 2002, ECOL APPL	10.2307/3060997	595			√
WEISS H, 1993, SCIENCE	10.1126/science.261.5124.995	573			√
MCGUIRE AD, 2001, GLOBAL BIOGEOCHEM CY	10.1029/2000GB001298	563		√	
GARCIA-RUIZ JM, 2011, EARTH-SCI REV	10.1016/j.earscirev.2011.01.006	551		√	
PAILLET Y, 2010, CONSERV BIOL	10.1111/j.1523-1739.2009.01399.x	547			√
LAURANCE WF, 2011, BIOL CONSERV	10.1016/j.biocon.2010.09.021	542			√
BROWN DG, 2005, ECOL APPL	10.1890/03-5220	534		√	
CRAMER VA, 2008, TRENDS ECOL EVOL	10.1016/j.tree.2007.10.005	528	√		
KRAUSS J, 2010, ECOL LETT	10.1111/j.1461-0248.2010.01457.x	505		√	
HOUGHTON RA, 2000, NATURE	10.1038/35002062	499		√	

Paper	DOI	Total Citations	Main research	Appears as a marginal study (appears as a sub study of other studies, or is only mentioned)	The study was not mentioned (the study on abandonment of farmland was not carried out)
STEHFEST E, 2009, CLIMATIC CHANGE	10.1007/s10584-008-9534-6	490			√
MOREIRA F, 2011, J ENVIRON MANAGE	10.1016/j.jenvman.2011.06.028	488			√
DUPOUEY JL, 2002, ECOLOGY	10.1890/0012-9658(2002)083[2978:IIOPLU]2.0.CO;2	487	√		
FIELD CB, 2008, TRENDS ECOL EVOL	10.1016/j.tree.2007.12.001	465		√	
FOSTER DR, 1992, J ECOL	10.2307/2260864	452			√
POSCHLOD P, 2002, BIOL CONSERV	10.1016/S0006-3207(01)00201-4	452			√
VERBURG PH, 2009, LANDSCAPE ECOL	10.1007/s10980-009-9355-7	448	√		
FUKAMI T, 2005, ECOL LETT	10.1111/j.1461-0248.2005.00829.x	448		√	
GARCIA-RUIZ JM, 2010, CATENA	10.1016/j.catena.2010.01.001	435		√	
QADIR M, 2014, NAT RESOUR FORUM	10.1111/1477-8947.12054	432			√
GEHRIG-FASEL J, 2007, J VEG SCI	10.1658/1100-9233(2007)18[571:TLSITS]2.0.CO;2	431			√
SCHIMEL D, 2000, SCIENCE	10.1126/science.287.5460.2004	430		√	
SCHEIDEGGER Y, 2000, OECOLOGIA	10.1007/s004420000466	428			√
HENLE K, 2008, AGR ECOSYST ENVIRON	10.1016/j.agee.2007.09.005	420		√	

Paper	DOI	Total Citations	Main research	Appears as a marginal study (appears as a sub study of other studies, or is only mentioned)	The study was not mentioned (the study on abandonment of farmland was not carried out)
CAMPBELL JE, 2008, ENVIRON SCI TECHNOL	10.1021/es800052w	417	√		
KOWALCHUK GA, 2002, ANTON LEEUW INT J G	10.1023/A:1020565523615	405			√
SHAKESBY RA, 2011, EARTH-SCI REV	10.1016/j.earscirev.2011.01.001	395		√	
SERRA P, 2008, APPL GEOGR	10.1016/j.apgeog.2008.02.001	394		√	
NAVARRO LM, 2012, ECOSYSTEMS	10.1007/s10021-012-9558-7	384	√		
SCHILLING J, 2008, J AM PLANN ASSOC	10.1080/01944360802354956	384			√
HOLL KD, 2000, RESTOR ECOL	10.1046/j.1526-100x.2000.80049.x	382			√
AIDE TM, 2000, RESTOR ECOL	10.1046/j.1526-100x.2000.80048.x	374			√
CHAZDON RL, 2007, PHILOS T R SOC B	10.1098/rstb.2006.1990	372		√	
FINEGAN B, 1996, TRENDS ECOL EVOL	10.1016/0169-5347(96)81090-1	372		√	
TSCHARNTKE T, 2011, J APPL ECOL	10.1111/j.1365-2664.2010.01939.x	371			√
ROUNSEVELL MDA, 2006, AGR ECOSYST ENVIRON	10.1016/j.agee.2005.11.027	370		√	

Paper	DOI	Total Citations	Main research	Appears as a marginal study (appears as a sub study of other studies, or is only mentioned)	The study was not mentioned (the study on abandonment of farmland was not carried out)
MEYFROIDT P, 2011, ANNU REV ENV RESOUR	10.1146/annurev-environ-090710-143732	363			√
BIGNAL EM, 1996, J APPL ECOL	10.2307/2404973	361			√
RUDEL TK, 2009, P NATL ACAD SCI USA	10.1073/pnas.0812540106	357		√	
HURTT GC, 2006, GLOBAL CHANGE BIOL	10.1111/j.1365-2486.2006.01150.x	352		√	
SILVER WL, 2000, RESTOR ECOL	10.1046/j.1526-100x.2000.80054.x	345		√	
MOTTET A, 2006, AGR ECOSYST ENVIRON	10.1016/j.agee.2005.11.017	344			√
FLINN KM, 2005, FRONT ECOL ENVIRON	10.1890/1540-9295(2005)003[0243:ROFPCI]2.0.CO;2	343		√	
GELLRICH M, 2007, AGR ECOSYST ENVIRON	10.1016/j.agee.2006.05.001	342	√		
KAPLAN JO, 2011, HOLOCENE	10.1177/0959683610386983	341			√
VERBURG PH, 2009, J ENVIRON MANAGE	10.1016/j.jenvman.2008.08.005	331			√
FISCHER M, 1997, CONSERV BIOL	10.1046/j.1523-1739.1997.96082.x	327			√
MATHER AS, 1998, AREA	10.1111/j.1475-4762.1998.tb00055.x	326		√	
ROSELL F, 2005, MAMMAL REV	10.1111/j.1365-2907.2005.00067.x	320			√

Paper	DOI	Total Citations	Main research	Appears as a marginal study (appears as a sub study of other studies, or is only mentioned)	The study was not mentioned (the study on abandonment of farmland was not carried out)
SOARES BS, 2002, ECOL MODEL	10.1016/S0304-3800(02)00059-5	319		√	
WHEELER BD, 2000, J ECOL	10.1046/j.1365-2745.2000.00455.x	312			√
DAVIDSON EA, 2007, NATURE	10.1038/nature05900	310		√	
TASSER E, 2007, AGR ECOSYST ENVIRON	10.1016/j.agee.2006.05.004	309	√		
DENGLER J, 2014, AGR ECOSYST ENVIRON	10.1016/j.agee.2013.12.015	308			√
LIM HS, 2008, J GEOCHEM EXPLOR	10.1016/j.gexplo.2007.04.008	307			√
TASSER E, 2002, APPL VEG SCI	10.1658/1402-2001(2002)005[0173:IOLUCO]2.0.CO;2	306			√
STOFFLER D, 2001, SPACE SCI REV	10.1023/A:1011937020193	305			√
DIRNBOCK T, 2003, J BIOGEOGR	10.1046/j.1365-2699.2003.00839.x	300			√
VAN VLIET J, 2015, LANDSCAPE URBAN PLAN	10.1016/j.landurbplan.2014.09.001	296		√	
PIMENTEL D, 1998, ECOSYSTEMS	10.1007/s100219900035	292		√	
CHAZDON RL, 2016, SCI ADV	10.1126/sciadv.1501639	292			√
GARCIA-RUIZ JM, 2011, AGR ECOSYST ENVIRON	10.1016/j.agee.2011.01.003	287	√		
STRIJKER D, 2005, BASIC APPL ECOL	10.1016/j.baae.2005.01.001	286		√	

Paper	DOI	Total Citations	Main research	Appears as a marginal study (appears as a sub study of other studies, or is only mentioned)	The study was not mentioned (the study on abandonment of farmland was not carried out)
BAUMANN M, 2011, LAND USE POLICY	10.1016/j.landusepol.2010.11.003	285	√		
HOOGWIJK M, 2005, BIOMASS BIOENERG	10.1016/j.biombioe.2005.05.002	282		√	
VIEIRA DLM, 2006, RESTOR ECOL	10.1111/j.1526-100X.2006.00100.x	281			√
MORRIEN E, 2017, NAT COMMUN	10.1038/ncomms14349	276		√	
RICE RA, 2000, AMBIO	10.1579/0044-7447-29.3.167	275			√
NEPSTAD DC, 2002, J GEOPHYS RES-ATMOS	10.1029/2001JD000360	274			√
GIBBS HK, 2015, APPL GEOGR	10.1016/j.apgeog.2014.11.024	273		√	
HERMY M, 2007, ECOL RES	10.1007/s11284-007-0354-3	273		√	
CAI XM, 2011, ENVIRON SCI TECHNOL	10.1021/es103338e	272		√	
JANGID K, 2011, SOIL BIOL BIOCHEM	10.1016/j.soilbio.2011.06.022	272			√
FUKAMI T, 2005, P ROY SOC B-BIOL SCI	10.1098/rspb.2005.3277	270		√	
MIAO YX, 2011, AGRON SUSTAIN DEV	10.1051/agro/2010034	270			√
KARDOL P, 2010, TRENDS ECOL EVOL	10.1016/j.tree.2010.09.001	267		√	

Paper	DOI	Total Citations	Main research	Appears as a marginal study (appears as a sub study of other studies, or is only mentioned)	The study was not mentioned (the study on abandonment of farmland was not carried out)
LASANTA-MARTINEZ T, 2005, APPL GEOGR	10.1016/j.apgeog.2004.11.001	266		√	
AIDE TM, 1995, FOREST ECOL MANAG	10.1016/0378-1127(95)03576-V	264			√
MAYAUX P, 2005, PHILOS T R SOC B	10.1098/rstb.2004.1590	264			√
DRUMMOND MA, 2010, BIOSCIENCE	10.1525/bio.2010.60.4.7	263		√	
QUEIROZ C, 2014, FRONT ECOL ENVIRON	10.1890/120348	261	√		
DOTTERWEICH M, 2008, GEOMORPHOLOGY	10.1016/j.geomorph.2008.05.023	261		√	
BUCKLEY DH, 2001, MICROB ECOL	10.1007/s002480000108	259		√	
RENWICK A, 2013, LAND USE POLICY	10.1016/j.landusepol.2012.04.005	256	√		
WILSON JD, 1997, J APPL ECOL	10.2307/2405262	254			√

Supplementary Table S3 Most Global Cited Documents 100 (title)

Paper	DOI	Total Citations	Main research	Appears as a marginal study (appears as a sub study of other studies, or is only mentioned)	The study was not mentioned (the study on abandonment of farmland was not carried out)
CRAMER VA, 2008, TRENDS ECOL EVOL	10.1016/j.tree.2007.10.005	531	√		
VERBURG PH, 2009, LANDSCAPE ECOL	10.1007/s10980-009-9355-7	449	√		
GEHRIG-FASEL J, 2007, J VEG SCI	10.1658/1100-9233(2007)18[571:TLSITS]2.0.CO;2	433			√
CAMPBELL JE, 2008, ENVIRON SCI TECHNOL	10.1021/es800052w	418	√		
SILVER WL, 2000, RESTOR ECOL	10.1046/j.1526-100x.2000.80054.x	347		√	
GELLRICH M, 2007, AGR ECOSYST ENVIRON	10.1016/j.agee.2006.05.001	342	√		
GARCIA-RUIZ JM, 2011, AGR ECOSYST ENVIRON	10.1016/j.agee.2011.01.003	288	√		
BAUMANN M, 2011, LAND USE POLICY	10.1016/j.landusepol.2010.11.003	287	√		
QUEIROZ C, 2014, FRONT ECOL ENVIRON	10.1890/120348	262	√		
RENWICK A, 2013, LAND USE POLICY	10.1016/j.landusepol.2012.04.005	259	√		
ESTEL S, 2015, REMOTE SENS ENVIRON	10.1016/j.rse.2015.03.028	252	√		
PRISHCHEPOV AV, 2013, LAND USE POLICY	10.1016/j.landusepol.2012.06.011	251	√		

Paper	DOI	Total Citations	Main research	Appears as a marginal study (appears as a sub study of other studies, or is only mentioned)	The study was not mentioned (the study on abandonment of farmland was not carried out)
POYATOS R, 2003, MT RES DEV	10.1659/0276-4741(2003)023[0362:LUALCC]2.0.CO;2	249		√	
ZHANG C, 2016, SOIL BIOL BIOCHEM	10.1016/j.soilbio.2016.02.013	249	√		
KOULOURI M, 2007, CATENA	10.1016/j.catena.2006.07.001	245	√		
LASANTA T, 2017, CATENA	10.1016/j.catena.2016.02.024	209	√		
LUGO AE, 2004, FOREST ECOL MANAG	10.1016/j.foreco.2003.09.012	207	√		
KUEMMERLE T, 2008, ECOSYSTEMS	10.1007/s10021-008-9146-z	206	√		
BOWEN ME, 2007, BIOL CONSERV	10.1016/j.biocon.2007.08.012	194		√	
ROMERO-CALCERRADA R, 2004, LANDSCAPE URBAN PLAN	10.1016/S0169-2046(03)00112-9	189	√		
GELLRICH M, 2007, LANDSCAPE URBAN PLAN	10.1016/j.landurbplan.2006.03.004	185	√		
VAN DER WAL A, 2006, SOIL BIOL BIOCHEM	10.1016/j.soilbio.2005.04.017	184	√		
SUAREZ-SEOANE S, 2002, BIOL CONSERV	10.1016/S0006-3207(01)00213-0	167	√		
MULLER D, 2013, AGR SYST	10.1016/j.agsy.2012.12.010	162	√		
HOOPER E, 2005, J APPL ECOL	10.1111/j.1365-2664.2005.01106.x	159		√	

Paper	DOI	Total Citations	Main research	Appears as a marginal study (appears as a sub study of other studies, or is only mentioned)	The study was not mentioned (the study on abandonment of farmland was not carried out)
ALCANTARA C, 2013, ENVIRON RES LETT	10.1088/1748-9326/8/3/035035	153	√		
LASANTA T, 2000, CATENA	10.1016/S0341-8162(99)00079-X	152	√		
DUNJO G, 2003, CATENA	10.1016/S0341-8162(02)00148-0	150	√		
CAMMERAAT LH, 1999, CATENA	10.1016/S0341-8162(98)00072-1	149		√	
BEILIN R, 2014, LAND USE POLICY	10.1016/j.landusepol.2013.07.003	149	√		
PRISHCHEPOV AV, 2012, ENVIRON RES LETT	10.1088/1748-9326/7/2/024021	148	√		
CHAUCHARD S, 2007, ECOSYSTEMS	10.1007/s10021-007-9065-4	147			√
GOUGH MW, 1990, BIOL CONSERV	10.1016/0006-3207(90)90104-W	147			√
HUNZIKER M, 1995, LANDSCAPE URBAN PLAN	10.1016/0169-2046(95)93251-J	146	√		
HEDLUND K, 2003, OIKOS	10.1034/j.1600-0706.2003.12511.x	146	√		
TERRES JM, 2015, LAND USE POLICY	10.1016/j.landusepol.2015.06.009	142	√		
ZHANG Y, 2014, LAND USE POLICY	10.1016/j.landusepol.2014.05.011	140	√		
SLUITER R, 2007, LANDSCAPE ECOL	10.1007/s10980-006-9049-3	136	√		
HOOPER E, 2002, ECOL APPL	10.1890/1051-0761(2002)012[1626:RONTST]2.0.CO;2	136		√	
LI SF, 2017, J GEOGR SCI	10.1007/s11442-017-1426-0	134	√		

Paper	DOI	Total Citations	Main research	Appears as a marginal study (appears as a sub study of other studies, or is only mentioned)	The study was not mentioned (the study on abandonment of farmland was not carried out)
LESSCHEN JP, 2008, EARTH SURF PROC LAND	10.1002/esp.1676	132	√		
WANG B, 2011, ENVIRON EARTH SCI	10.1007/s12665-010-0577-4	130	√		
KUEMMERLE T, 2011, GLOBAL CHANGE BIOL	10.1111/j.1365-2486.2010.02333.x	129	√		
MOLINILLO M, 1997, ENVIRON MANAGE	10.1007/s002679900051	123	√		
LABRECQUE M, 2003, BIOMASS BIOENERG	10.1016/S0961-9534(02)00192-7	123			√
LIU Y, 2012, GEOMORPHOLOGY	10.1016/j.geomorph.2011.10.009	121	√		
PLIENINGER T, 2014, PLOS ONE	10.1371/journal.pone.0098355	121	√		
ARNAEZ J, 2011, LAND DEGRAD DEV	10.1002/ldr.1032	121	√		
SCHIERHORN F, 2013, GLOBAL BIOGEOCHEM CY	10.1002/2013GB004654	120	√		
XU DD, 2019, J ENVIRON MANAGE	10.1016/j.jenvman.2018.11.136	119	√		
SIRAMI C, 2008, BIOL CONSERV	10.1016/j.biocon.2007.10.015	119	√		
CERDA A, 1997, ARID SOIL RES REHAB	10.1080/15324989709381469	116		√	

Paper	DOI	Total Citations	Main research	Appears as a marginal study (appears as a sub study of other studies, or is only mentioned)	The study was not mentioned (the study on abandonment of farmland was not carried out)
DIAZ GI, 2011, LANDSCAPE URBAN PLAN	10.1016/j.landurbplan.2010.11.005	114	√		
MUNROE DK, 2013, CURR OPIN ENV SUST	10.1016/j.cosust.2013.06.010	110	√		
UCHIDA K, 2014, ECOL MONOGR	10.1890/13-2170.1	109	√		
HOOGWIJK M, 2009, BIOMASS BIOENERG	10.1016/j.biombioe.2008.04.005	107		√	
LESSCHEN JP, 2007, CATENA	10.1016/j.catena.2006.05.014	105	√		
CHAPMAN CA, 1999, CONSERV BIOL	10.1046/j.1523-1739.1999.98229.x	104		√	
YAN JZ, 2016, LAND USE POLICY	10.1016/j.landusepol.2016.06.014	102	√		
HATNA E, 2011, ECOSYSTEMS	10.1007/s10021-011-9441-y	101		√	
HOLTKAMP R, 2008, APPL SOIL ECOL	10.1016/j.apsoil.2007.11.002	101	√		
ZELLER V, 2001, SOIL BIOL BIOCHEM	10.1016/S0038-0717(00)00208-X	101			√
SIRAMI C, 2007, DIVERS DISTRIB	10.1111/j.1472-4642.2006.00297.x	98		√	
PRISHCHEPOV AV, 2012, REMOTE SENS ENVIRON	10.1016/j.rse.2012.08.017	97	√		

Paper	DOI	Total Citations	Main research	Appears as a marginal study (appears as a sub study of other studies, or is only mentioned)	The study was not mentioned (the study on abandonment of farmland was not carried out)
DUARTE F, 2008, J ENVIRON MANAGE	10.1016/j.jenvman.2007.05.024	95	√		
MEYFROIDT P, 2016, GLOBAL ENVIRON CHANG	10.1016/j.gloenvcha.2016.01.003	94	√		
LEVERS C, 2018, SCI TOTAL ENVIRON	10.1016/j.scitotenv.2018.06.326	94	√		
CHERRY DS, 2001, ENVIRON POLLUT	10.1016/S0269-7491(00)00093-2	94			√
GIBSON CWD, 1987, BIOL CONSERV	10.1016/0006-3207(87)90132-7	94		√	
YIN H, 2018, REMOTE SENS ENVIRON	10.1016/j.rse.2018.02.050	93	√		
BENJAMIN K, 2005, LANDSCAPE ECOL	10.1007/s10980-005-0068-2	91	√		
PREVOSTO B, 2011, FOLIA GEOBOT	10.1007/s12224-010-9096-z	91		√	
ZHANG KR, 2010, FOREST ECOL MANAG	10.1016/j.foreco.2010.02.014	91	√		
BENAYAS JMR, 2005, FOREST ECOL MANAG	10.1016/j.foreco.2005.03.032	90	√		
ROMERO-DIAZ A, 2017, CATENA	10.1016/j.catena.2016.08.013	87	√		
ZHANG JT, 2005, J ARID ENVIRON	10.1016/j.jaridenv.2005.03.027	87	√		
PICHTEL JR, 1994, J ENVIRON QUAL	10.2134/jeq1994.00472425002300040022x	86			√

Paper	DOI	Total Citations	Main research	Appears as a marginal study (appears as a sub study of other studies, or is only mentioned)	The study was not mentioned (the study on abandonment of farmland was not carried out)
SANCHEZ-CUERVO AM, 2013, ECOSYSTEMS	10.1007/s10021-013-9667-y	86	√		
SPERA SA, 2014, ENVIRON RES LETT	10.1088/1748-9326/9/6/064010	85			√
PAZUR R, 2014, APPL GEOGR	10.1016/j.apgeog.2014.07.014	83	√		
JOHANSSON T, 1999, BIOMASS BIOENERG-a	10.1016/S0961-9534(98)00075-0	82	√		
WANG B, 2013, EARTH SURF PROC LAND	10.1002/esp.3459	81	√		
LESSCHEN JP, 2008, J ARID ENVIRON	10.1016/j.jaridenv.2008.06.006	81	√		
GARCIA-RUIZ JM, 2005, CATENA	10.1016/j.catena.2004.05.006	80		√	
CERDA A, 2018, PROG PHYS GEOG	10.1177/0309133318758521	80	√		
COLON SM, 2006, BIOTROPICA	10.1111/j.1744-7429.2006.00159.x	79		√	
HOOPER ER, 2004, ECOLOGY	10.1890/03-0655	79			√
KAUFFMAN JB, 2009, ECOL APPL	10.1890/08-1696.1	79		√	
HARMER R, 2001, BIOL CONSERV	10.1016/S0006-3207(01)00072-6	79	√		
XIE HL, 2014, SUSTAINABILITY-BASEL	10.3390/su6031260	79	√		

Paper	DOI	Total Citations	Main research	Appears as a marginal study (appears as a sub study of other studies, or is only mentioned)	The study was not mentioned (the study on abandonment of farmland was not carried out)
NOVARA A, 2016, SCI TOTAL ENVIRON	10.1016/j.scitotenv.2016.01.095	79	√		
NOVARA A, 2017, SCI TOTAL ENVIRON	10.1016/j.scitotenv.2016.10.123	78	√		
NADAL-ROMERO E, 2016, AGR ECOSYST ENVIRON	10.1016/j.agee.2016.05.003	77	√		
CORBELLE-RICO E, 2012, LAND USE POLICY	10.1016/j.landusepol.2011.08.008	77	√		
HARDEN CP, 1996, MT RES DEV	10.2307/3673950	76	√		
GISPERT M, 2013, GEODERMA	10.1016/j.geoderma.2013.03.012	76		√	
PRICE B, 2015, APPL GEOGR	10.1016/j.apgeog.2014.12.009	75		√	
SIKOR T, 2009, WORLD DEV	10.1016/j.worlddev.2008.08.013	74	√		
VASSILEV K, 2011, PLANT BIOSYST	10.1080/11263504.2011.601337	74	√		
SHANG ZH, 2008, LAND DEGRAD DEV	10.1002/ldr.861	74		√	
